# A Periosteum‐Inspired Janus Piezoelectric Scaffold Using Bioenergetic‐Driven H‐Type Vascularization for Diabetic Bone Regeneration

**DOI:** 10.1002/advs.75948

**Published:** 2026-06-04

**Authors:** Kai Wang, Kai Jiang

**Affiliations:** ^1^ Department of Spine Surgery Honghui Hospital Xi'an Jiaotong University Xi'an Shanxi China; ^2^ Department of Critical Care Medicine Sichuan Provincial People's Hospital University of Electronic Science and Technology of China Chengdu China

**Keywords:** diabetic bone regeneration, organoid, periosteum, piezoelectricity, vascularization

## Abstract

An imbalance in the bone–vessel‐coupled regeneration process is one of the key factors hindering the effective treatment of diabetic bone defects, which are rooted in the impaired function of mitochondria. Inspired by the bilayered heterogeneous structure of the periosteum, a bilayered, biomimetic, Janus piezoelectric scaffold (JPS) with endogenous piezoelectric effects was developed to regulate energy metabolism. JPS comprises a zinc‐doped apatite–gelatin–poly‐L‐lactic acid membrane fabricated using electrospinning and integrated with a resveratrol‐loaded microsphere composite hydrogel. Without requiring an external power source, JPS is designed to respond to local micromechanical stimulation and generate electroactive cues, while synergizing with the controlled release of resveratrol to regulate angiogenesis‐related signaling and endothelial metabolic reprogramming. Single‐cell sequencing indicated that bone microenvironment remodeling may be associated with H‐type vessels, which are bone‐specific microvasculatures. JPS provides a continuous power source in a high‐sugar environment, thereby reconstructing the bone–vessel coupled regeneration process. JPS treatment stimulated the vascular features associated with H‐type vessel‐mediated bone regeneration in vascular organoids. The JPS group showed a substantially enhanced bone volume of 21.1% vs. 8.5% in the control in a diabetic calvarial defect model. This study presents an efficient material platform for addressing clinical challenges associated with diabetic bone regeneration.

## Graphical abstract

A periosteum‐inspired Janus piezoelectric scaffold converts micromechanical forces into electrical signals and synergizes with controlled resveratrol release. This dual action reprograms endothelial metabolism, drives H‑type vessel formation, and restores bone–vessel coupling in diabetic bone defects.

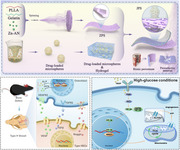

## 1 Introduction

The disruption of the bone–vascular coupling mechanism is one of the central challenges in the treatment of diabetic bone defects [1, 2]. The restoration of normal physiological function in newly‐formed bone relies on adequate angiogenesis [3, 4]. Under diabetic pathological conditions, the sustained hyperglycemic microenvironment directly disrupts adenosine triphosphate metabolic homeostasis and induces structural and functional impairments in endothelial cell mitochondria [5, 6]. The resulting energy deficiency leads to delayed or failed vascular repair responses, ultimately compromising the spontaneous bone regeneration process. This pathological mechanism has become a critical limiting factor that restricts existing bone repair materials from achieving the expected therapeutic outcomes in the treatment of diabetic bone defects [7, 8]. A prevailing limitation in numerous studies is the tendency to investigate the promotive effects of bone repair materials on vascular repair or osteogenesis in isolation, while neglecting the driving role of energy metabolism [9, 10, 11]. Thus, revisiting bone repair materials from the perspective of energy metabolism to elucidate the imbalance in the bone–vascular coupling mechanism is a highly promising and necessary strategy for promoting effective regeneration of diabetic bone defects.

The periosteum is a bilayered, heterogeneous organ composed of an outer fibrous layer and an inner cambium layer, providing an excellent blueprint for the design of biomimetic bone‐repair materials [12, 13, 14]. From a compositional and structural perspective, the outer layer of the periosteum consists of a dense network of collagen fibers arranged in an interwoven three‐dimensional structure [15, 16]. This structure can effectively respond to mechanical stress and generate tiny electric charges, thus creating an electric field [17, 18]. The inner layer of the periosteum is composed of numerous bone cells, blood vessels, and bone matrix and serves as a crucial site for initiating bone repair [19, 20]. Due to their dual‐faced, heterogeneous characteristics, Janus materials are an ideal choice for mimicking the periosteum [21, 22]. Compared with conventional bilayer scaffolds with weak interfacial adhesion between simply stacked layers, the Janus scaffold offers unique advantages, including strong interfacial integration, dual‐functionality within a single architecture, gradient continuity that mimics native tissue interfaces, and controllable directional release of bioactive drugs. These features enable more coordinated osteogenesis–angiogenesis coupling and improved structural stability during bone regeneration. The photo‐crosslinked, strontium‐substituted, hydroxyapatite‐mineralized, type I collagen Janus membrane has been successful in promoting bone marrow mesenchymal stromal cell (BMSC) homing and angiogenesis [23]. Similarly, collagen membranes based on a Janus structure with a porous layer can effectively promote the direct differentiation and inward migration of osteoblasts. The dense layer acts as a barrier to prevent soft tissue infiltration into the bone defect site, thereby providing a protected space for osteogenesis [24]. Current research on Janus materials has successfully achieved an effective imitation of the geometric features of periosteal structures. However, further investigation is needed to explore the physiological functions and microelectric field effects of biomimetic periosteum materials in living organisms.

The endogenous piezoelectric effect is an important signaling mechanism during bone remodeling and plays a key role in regulating the balance between bone resorption and regeneration [25, 26]. When micromotion occurs at the fracture ends, the organism spontaneously converts mechanical energy into electrical signals, which subsequently stimulate the proliferation and differentiation of osteoblasts, resulting in the formation of new bone tissue [27, 28, 29]. Sintered whitlockite nanoparticles were incorporated into the system to endow the double‐network hydrogel composed of chelated alginate and gelatin methacrylate with piezoelectric properties. Improvement in the bioelectric microenvironment promoted bone repair [14]. Using vacuum filtration, nanoparticles assembled from components of piezoelectric material, K_0.5_Na_0.5_NbO_3_ (KNN) and saikosaponin D (SSD), were embedded into a decellularized adipose tissue matrix, successfully constructing an ultrasound‐responsive piezoelectric composite membrane. Under ultrasound irradiation, the membrane enabled controlled piezoelectric stimulation and SSD release, facilitating the regeneration of osteoporotic bone defects without infection risks associated with exogenous electrical stimulation [30]. Incorporating piezoelectric nanoparticles into materials is an effective strategy for enhancing piezoelectric performance [31, 32, 33]. Furthermore, the piezoelectric effect should be regarded as a core consideration in the design and fabrication of future bone‐repair scaffolds.

**FIGURE 1:**
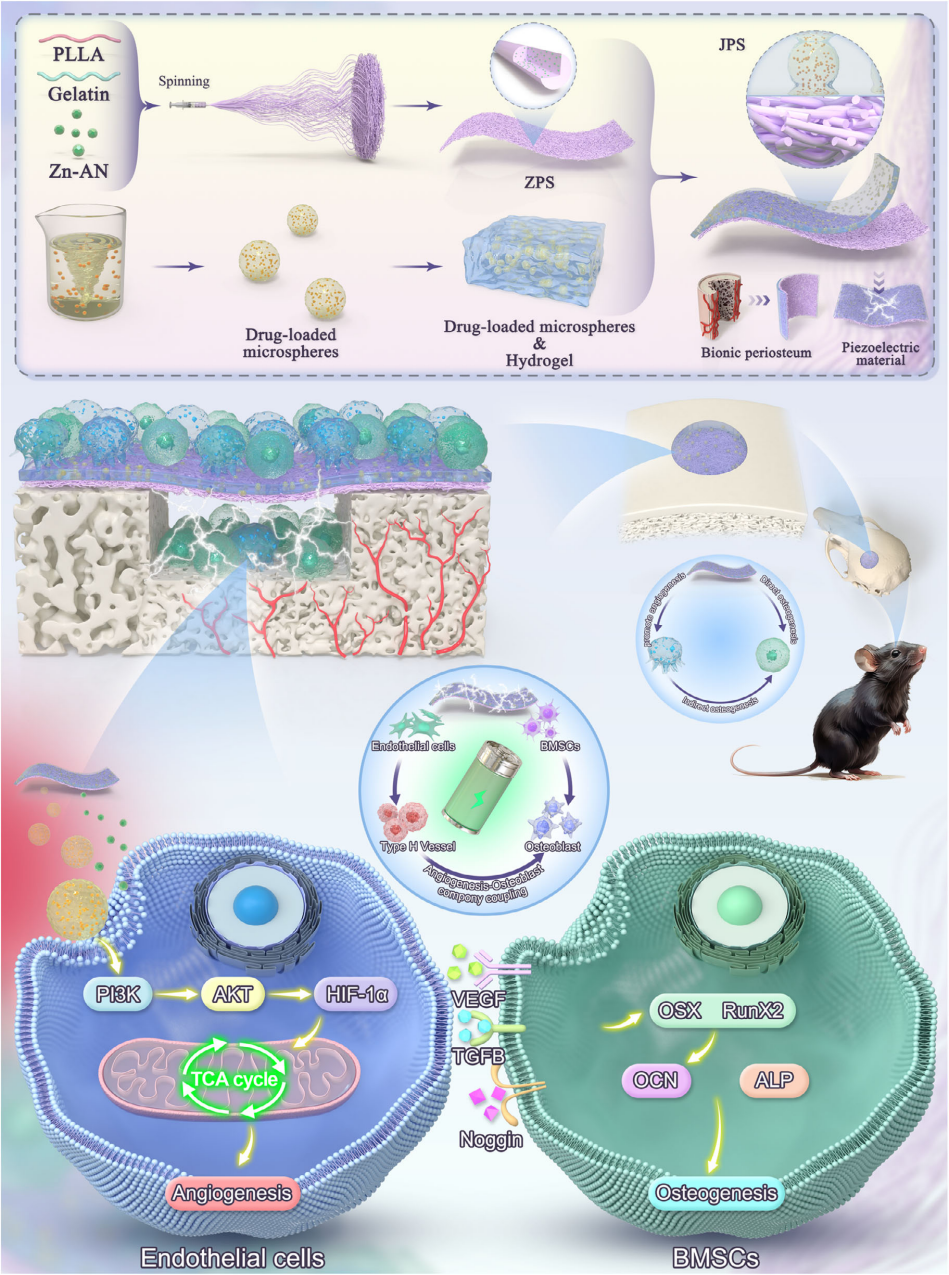
Fabrication schematic of periosteum‐inspired Janus piezoelectric scaffold (JPS) for diabetic bone regeneration. The JPS, featuring a biomimetic bilayered structure, is designed to improve the diabetic regenerative microenvironment through the synergy between its piezoelectric responsiveness and bioactive molecule release. This design is intended to favor H‐type vessel–associated vascularization, which supports angiogenesis and osteogenesis, thereby facilitating coupled bone regeneration under diabetic conditions.

Inspired by the bilayer heterogeneous structure of the periosteum, a Janus piezoelectric scaffold (JPS) was developed, which utilized the micromechanical energy of fractures to generate a piezoelectric effect. A piezoelectric response drug release coupling strategy was developed by integrating a hydrogel composite loaded with resveratrol microspheres, which effectively promotes the repair of diabetic bone defects (Figure 1). JPS is composed of a gelatin–poly‐L‐lactic acid (PLLA) membrane fabricated via electrospinning and a drug‐loaded microsphere composite hydrogel. A genipin‐mediated chemical cross‐linking strategy served to autonomously and firmly integrate the two layers at the interface. Upon zinc‐apatite doping, the gelatin–PLLA membrane demonstrated a stable and responsive piezoelectric signal while concurrently releasing Zn^2+,^ which promoted angiogenesis. The composite hydrogel layer possesses excellent biocompatibility and achieves precise release of resveratrol. This coupled piezoelectric response drug release strategy operated via a dual mechanism. The cascade of the voltage‐gated channel‐associated signaling pathway was amplified, and the precise release of mitochondria‐targeting resveratrol was enabled, which promoted glucose uptake and specifically induced the formation of H‐type blood vessels. This strategy acts as an energy‐control scaffold for the differentiation and maturation of endothelial cells, guaranteeing the effective regeneration of diabetic bone defects from the perspective of energy supply.

## 2 Materials and Methods

### 2.1 Materials

Carboxymethyl chitosan (Degree of substitution: ≥80%), PLLA (M_n_5000), ZnCl_2_, NaCl, NaHCO_3_, KCl, K_2_HPO_4_∙3H_2_O, MgCl_2_∙6H_2_O, CaCl_2_, and Na_2_SO_4_ were obtained from Aladdin, Shanghai, China. Acetic acid, Glutaraldehyde (25%), and Gelatin were purchased from Chengdu Kelong Chemical Reagent Factory, China. Genipin was acquired from Linchuan Zhixin Bio‐Technology Co., Ltd., China. Resveratrol (Res, purity ≥ 99%) was purchased from Shanghai Macklin Biochemical Technology Co., Ltd, China. The CCK‐8 kit and Calcein‐AM/PI double staining were supplied by Beyotime Biotechnology (China). DMEM/F12 and MEM‐α complete media, supplemented with 10% fetal bovine serum and 1% penicillin‐streptomycin, as well as trypsin, were purchased from Gibco (America).

### 2.2 Sample Preparation

Resveratrol (200 mg) was added to 100 mL of a 3% (w/v) carboxymethyl chitosan solution, thoroughly stirred, and then allowed to stand overnight. 10 mL liquid paraffin was measured and mixed with 0.2 mL Span‐80, followed by stirring at 50°C for 10 min at 1200 rpm. The resveratrol/carboxymethyl chitosan mixture was then slowly dripped into the solution and emulsified at 50°C. Subsequently, 0.2 mL of glutaraldehyde solution was added to the system, and stirring was continued for 1 h to facilitate the cross‐linking of carboxymethyl chitosan. Subsequently, twice the volume of anhydrous ethanol was added to the mixture, followed by demulsification in an ice‐water bath for 30 min at 1200 rpm. The sample was then centrifuged, and the gel layer was collected to obtain resveratrol‐loaded carboxymethyl chitosan microspheres.

PLLA (1 g) and gelatin (0.6 g) were dissolved in 10 g of hexafluoroisopropanol, and after stirring for 12 h, an electrospinning solution was obtained. Piezoelectric scaffolds (PS) were fabricated via electrospinning under the following conditions: a voltage of 17 kV, a constant temperature of 28°C, a humidity of 40%, and a collection distance of 15 cm. 100 mg of ZnCl_2_ was added to the 4 × SBF solution (Table S1), followed by incubation at 37°C for 48 h. The mixture was then centrifuged to collect white mineralized particles. Subsequently, the mixture was freeze‐dried for 3 d to obtain zinc‐doped apatite nanoparticles (Zn‐AN). 20 mg of Zn‐AN was added to the aforementioned spinning solution, and zinc‐doped piezoelectric scaffolds (ZPS) were fabricated using the identical experimental procedure.

ZPS was immersed in 20 mL of a 0.1% (w/v) Genipin solution until fully saturated. Resveratrol‐loaded carboxymethyl chitosan microspheres were thoroughly mixed with a 3% carboxymethyl chitosan solution. The resulting mixture was then coated onto one side of the ZPS, and the obtained composite scaffold was designated as Janus piezoelectric scaffolds (JPS).

### 2.3 Sample Characterization

Physical and chemical properties: Field‐emission scanning electron microscopy (FE‐SEM; ZEISS Gemini SEM 300, Germany) was employed to examine the surface morphology and cross‐sectional characteristics of the scaffolds. X‐ray energy‐dispersive spectroscopy (EDS; OXFORD Xplore, UK) was used to perform elemental mapping of C, O, Ca, P, Zn, and N of samples. The crystal structure of scaffolds was analyzed by using x‐ray diffraction analysis (XRD; Rigaku SmartLab SE, Japan) in the range of 10°–80°. The lattice arrangement of samples was examined using transmission electron microscopy (TEM; JEOL JEM‐F200, Japan). Fourier transform infrared spectroscopy (FT‐IR; Thermo Nicolet IS5, USA) was performed with a wavenumber range of 550–2000 cm−^1^. The Ca^2+^ and Zn^2+^ release rate of JPS was tested via an inductively coupled plasma (ICP) emission spectrometer (Agilent 5100 SVDV, USA). The drug release mechanism was analyzed via a UV–vis spectrophotometer (UV–vis; Lambda 750 s, USA), and the test wavelength is 307 nm.

Piezoelectric performance: The piezoelectric constants of all scaffolds were tested using a quasi‐static d33 meter (ZJ‐3, China). Butterfly loop, hysteresis loop, topography, amplitude image, and phase image were obtained via using piezo‐response force microscopy (PFM; Bruker MultiMode8, Germany). The piezoelectric scaffold is clamped between two copper foils as counter electrodes, with leads connected by soldering to the copper electrodes. The structure is then encapsulated using polyimide (PI) film to secure the assembly and provide insulation. During testing, the fabricated device is connected to a charge signal conditioning and acquisition circuit. A 2N steady‐state or dynamic force is applied to the device surface, and the output voltage signal is recorded via an AD7608 multi‐channel synchronous acquisition chip. The voltage response curve is ultimately analyzed to evaluate the piezoelectric performance.

### 2.4 Single‐Cell Sequencing Analysis

Single‐cell RNA sequencing (scRNA‐seq) data of bone tissue from Type 2 diabetes mellitus (T2M) mice and normal control mice were downloaded from the public database Gene Expression Omnibus (GEO). The raw gene expression matrix was processed using the Seurat R package (version 4.0.0). Data were normalized using the NormalizeData function. Major cell types were manually annotated based on well‐established marker genes. Visualization of the significantly differentially expressed genes in endothelial cells was achieved with a volcano plot. KEGG pathway and GO analyses were performed on the differentially expressed genes using the clusterProfiler R package, with a *p*‐value < 0.05 considered statistically significant. Next, cell communication between the osteoblast lineage and endothelial cells was inferred using the CellChat R package. Communication probabilities and interaction strengths were calculated based on a curated ligand‐receptor interaction database. Finally, the interaction networks and the number of inferred ligand‐receptor pairs were compared between the T2M and control groups.

### 2.5 Evaluation of Biocompatibility

Human umbilical vein endothelial cells (HUVECs) were purchased from the Cell Bank of the Chinese Academy of Sciences. In this study, HUVECs were used as a standard in vitro endothelial model to evaluate the general angiogenic response of endothelial cells to different scaffold treatments under high‐glucose conditions, rather than as a direct cellular substitute for bone‐specific type H vessels. Primary bone marrow‐derived mesenchymal stem cells (BMSCs) were isolated from 6‐week‐old C57BL/6 mice and expanded to passages 3–5 for subsequent experiments. The samples were sterilized by ultraviolet (UV) irradiation for 30 min per side, followed by three washes with PBS to remove potential impurities. Experimental extracts were prepared by immersing scaffolds (1 mL per 0.01 g of scaffolds) in a high‐glucose medium and incubating at 37°C for 24 h. Unless otherwise specified, the experimental groups were defined as follows: Control, normal‐glucose medium (5.5 mm glucose); HG, high‐glucose medium (25 mm glucose) without scaffold treatment; and HG+PS, HG+ZPS, and HG+JPS, the corresponding scaffold‐treated groups under the same high‐glucose condition. For short‐term endothelial assays, HUVECs were exposed to high glucose for 48 h before sample collection. For osteogenic assays, BMSCs in the HG‐related groups were maintained in high‐glucose medium throughout the indicated induction period.

BMSCs were co‐cultured with the sample extracts for 1, 3, and 5 d, followed by CCK‐8 assay. At each time point, the culture medium was aspirated, and 500 µL of fresh medium containing 10% CCK‐8 reagent was added, followed by incubation in the dark for 2 h. Subsequently, the OD value was measured at a wavelength of 450 nm using a microplate reader (BioTek, Synergy H1).

Cytoskeleton staining was performed after co‐culturing scaffolds with BMSCs for 3 d. Cell nuclei were fluorescently stained with DAPI, and cellular cytoskeletons were labeled with tubulin antibody staining, incubated at 37°C in the dark for 15–20 min. After washing with PBS, the samples were observed using a confocal laser scanning microscope (CLSM, Zeiss LSM 880).

The effect of the scaffold on BMSC recruitment was investigated using a scratch assay. After the BMSCs were cultured to form a 100% confluent monolayer, a scratch was made vertically in the center using a pipette tip. The dislodged cells were removed by washing with PBS, and the medium was replaced with serum‐free medium (without Fetal Bovine Serum) to inhibit cell proliferation. Images of the scratch area were captured at the identical location at 0 and 48 h using an inverted optical microscope (Nikon). The scratch width was measured using ImageJ software, and the cell migration rate was calculated.

Migration rate (%)  =  [(W_0_‐W_48_) / W_0_]  ×  100%

### 2.6 Evaluation of In Vitro Angiogenesis Capability

A tube formation assay was conducted to assess the general endothelial angiogenic capability. HUVECs were cultured under the Control, HG, HG+PS, HG+ZPS, and HG+JPS conditions. The Matrigel matrix was thawed overnight at 4°C. Then, 50 µL of the solution was added to a pre‐cooled 96‐well plate, followed by incubation at 37°C for 30 min for polymerization. Conditioned medium (supernatant) was collected after 24 h of co‐culture under the corresponding treatment conditions. HUVECs were then digested, resuspended, and seeded onto Matrigel‐coated 96‐well plates at a density of 2 × 10^4^ cells per well, followed by the addition of the corresponding conditioned medium. After 6 h of culture, tubular structure formation was observed using an inverted optical microscope (Nikon Eclipse Ti2). Quantitative analysis of the tube formation network was performed using the Angiogenesis Analyzer plugin in ImageJ software, including Total Tube Length, Number of Junctions, and Number of Meshes.

RT‐PCR analysis was further performed to detect the expression of angiogenesis‐associated genes. Total RNA was extracted from HUVECs in the Control, HG, HG+PS, HG+ZPS, and HG+JPS groups after 48 h of treatment using TRIzol. Then 1 µg of RNA was reverse‐transcribed into cDNA. The amplification procedure is as follows: 30 s of pre‐denaturation at 95°C; 5 s of denaturation at 95°C, 34 s of annealing/extension at 60°C, for a total of 40 cycles. The genes detected include vascular endothelial growth factor (VEGF), hypoxia‐inducible factor 1α (HIF‐1α), platelet‐derived growth factor (PDGF‐BB), and platelet‐endothelial cell adhesion molecule (CD31).

Finally, the immunofluorescence technique was employed to analyze the expression of endothelial angiogenesis‐related proteins. After 48 h of treatment under the corresponding glucose/scaffold conditions, the cells were washed twice with PBS and fixed with 4% paraformaldehyde for 15 min. The cells were permeabilized with PBS containing 0.1% Triton X‐100 for 15 min, followed by blocking with 1% BSA solution at room temperature for 1 h to prevent non‐specific binding. After removing the blocking solution, the primary antibody HIF‐1α (1:200 dilution) was added and incubated overnight at 4°C. The next day, FITC‐labeled secondary antibody (1:500) was added and incubated in the dark for 1 h. Finally, the nuclei were counterstained with DAPI solution for 5 min. The images were observed using a confocal laser scanning microscope (CLSM, Zeiss LSM 880). The average fluorescence intensity of HIF‐1α was semi‐quantitatively analyzed using ImageJ software.

### 2.7 Evaluation of In Vitro Osteogenic Capability

For osteogenic assays, BMSCs were assigned to the Control, HG, HG+PS, HG+ZPS, and HG+JPS groups. In the HG‐related groups, cells were maintained in high‐glucose medium (25 mm glucose) throughout the indicated induction period. ALP staining was used to evaluate the early osteogenic differentiation potential of scaffolds. BMSCs co‐cultured with scaffolds for 14 d were fixed with 4% paraformaldehyde. According to the instructions of the manufacturer, the BCIP/NBT substrate working solution was added and incubated protected from light. The reaction was terminated when purple precipitates became visible under the microscope.

ARS staining was used to evaluate the formation of late‐stage mineralization nodules. After 21 d of co‐culture between scaffolds and BMSCs, the cells were fixed with 4% paraformaldehyde for 30 m, followed by PBS washing. Then, 1% Alizarin Red S solution (pH 4.2) was added and incubated at room temperature for 20 m. The red mineralization nodules were photographed using an inverted light microscope. For further quantification, 10% Cetylpyridinium chloride (CPC) solution was added to each well to dissolve the bound dye, and the absorbance was measured at a wavelength of 562 nm.

The expression of osteogenesis‐related genes was analyzed using PCR technology, following the same procedures as previously described. The genes examined included OCN, OPN, COL‐1, and Runx‐2 (Table S2). Finally, immunofluorescence was employed to analyze the expression of osteogenesis‐related proteins, with procedures consistent with those described earlier. OCN was the protein used for detection.

### 2.8 Evaluation of In Vitro Vascular Organogenesis Capability

Human pluripotent stem cells (hPSCs) were first digested into single cells and seeded in ultra‐low attachment U‐bottom 96‐well plates to allow for aggregation and formation of 3D embryoid bodies (EB). Following EB were directed toward mesodermal differentiation, and subsequently further induced toward endothelial differentiation. After a period of culture, mature vascular organoids (VO) were formed. The JPS samples were sequentially processed with 75% ethanol, PBS solution, and UV irradiation for sterilization. Approximately samples were gently mixed on ice and stored at 4°C for 12 h. Then, 40 µL Matrigel (356230, Corning) was added and gently mixed on ice before storing at 37°C for 30 min to obtain JPS@Matrigel. We used vessel organoids grown without JPS@Matrigel treatment as a control group. On 10, 20, and 30 d of culture, vascular organoids were subjected to live imaging using an inverted optical microscope (Nikon Eclipse Ti2). The overall morphology, sprouting, and branching of the vascular networks were documented, and the maximum diameter was quantitatively analyzed using ImageJ software.

After 30 d of culture, the organoids were collected and fixed overnight at 4°C using 4% paraformaldehyde. The organoids were then embedded in paraffin and sectioned into 5 µm thick slices. Standard H&E staining was performed, followed by observation and imaging under an optical microscope. Quantitative analyses of vascular density and average lumen diameter were conducted using ImageJ software. To further observe the vascular ultrastructure, the samples were sequentially processed through standard fixation, dehydration, embedding, and section staining procedures. Observation and imaging were then carried out using a Hitachi HT‐7800 transmission electron microscope, followed by quantitative analysis of endothelial cell junctions, basement membrane integrity, and the morphology of organelles.

Finally, the immunofluorescence technique was employed to assess angiogenesis in each group. After permeabilization and blocking, the frozen sections were subjected to double immunofluorescence staining using an anti‐CD31 antibody (an endothelial cell marker, red fluorescence) and an anti‐EMCN antibody (an endothelial marker associated with type H vessel‐like characteristics in bone, green fluorescence), followed by nuclear counterstaining with DAPI. Images were observed using a CLSM.

### 2.9 Mechanism of Angiogenesis Capability of Janus‐Type Scaffolds

For transcriptome analysis, HUVECs were cultured in normal‐glucose medium (Control, 5.5 mm glucose) or high‐glucose medium (HG, 25 mm glucose). For RNA‐seq, total RNA was extracted from HUVECs after 48 h of treatment in the HG and HG+JPS groups, with 3 replicates set for each group, using the Trizol method. The Illumina NovaSeq 6000 system (Illumina, San Diego, CA, USA) was used for sequencing. Gene expression quantification and differential expression analysis were performed using StringTie and DESeq2. The criteria for screening differentially expressed genes (DEGs) were |log_2_(FoldChange)| > 1 and adjusted p‐value (padj) < 0.05. Subsequently, GO functional enrichment analysis of DEGs was performed using the clusterProfiler R package, covering three categories: Biological Process (BP), Cellular Component (CC), and Molecular Function (MF). KEGG pathway enrichment analysis of DEGs was performed using clusterProfiler, with the threshold set at p.adjust < 0.05.

Western blotting (WB) and Seahorse glycolysis analysis were employed to validate the potential signaling pathways and metabolic phenotype. Total protein and Seahorse samples were prepared from HUVECs in the Control, HG, HG+JPS, and HG+JPS+LY294002 groups after 48 h of treatment. For WB analysis, total protein samples were prepared from HUVECs in the Control, HG, HG+JPS, and HG+JPS+LY294002 groups after 48 h of treatment. For Seahorse glycolysis analysis, HUVECs were divided into the HG, HG+JPS, and HG+JPS+LY294002 groups after 48 h of treatment. 200 µL of RIPA lysis buffer containing 1% PMSF was added to each well and incubated on ice for 30 min. The cell lysates were collected and centrifuged at 12 000 rpm at 4°C for 15 min. The samples were sequentially processed through protein quantification, SDS‐PAGE electrophoresis, membrane transfer, and blocking. Then the membrane was incubated overnight at 4°C with primary antibodies (against VEGFA, PI3K, HIF‐1α, and β‐actin). The next day, the membrane was incubated with a secondary antibody at room temperature for 1 h. After thorough washing with TBST, the membranes were exposed using a Bio‐Rad imaging system (USA).

For Seahorse glycolysis analysis, HUVECs were seeded into Seahorse XF cell culture microplates at an optimized density and cultured under the indicated treatment conditions. Before measurement, the culture medium was replaced with Seahorse XF assay medium, and the cells were equilibrated in a non‐CO_2_ incubator according to the manufacturer's instructions. The extracellular acidification rate (ECAR) was recorded to evaluate glycolytic activity, and the obtained values were normalized to cell number or total protein content.

### 2.10 Evaluation of In Vivo Diabetic Bone Regeneration Potential

All animal experiments were reviewed and approved by the Animal Ethics Committee of Xi'an Jiaotong University (XJTUAE20‐2102). Male genetically diabetic db mice (BKS‐db/db) carrying the diabetes gene mutation were used to establish the diabetic calvarial defect model. The diabetic phenotype was confirmed according to the supplier‐provided strain information and available blood glucose measurements before surgery. The animals were randomly divided into 4 groups: control group, PS, ZPS, and JPS group (*n* = 5). A critical‐size full‐thickness bone defect (3 mm in diameter) was surgically created and subsequently filled with the implant scaffolds. The mice specimens were scanned using high‐resolution micro‐CT (Quantum GX II). The evaluated parameters included bone volume/tissue volume (BV/TV, %), trabecular number (Tb.N, 1/mm), trabecular thickness (Tb.Th, mm), and trabecular separation (Tb.Sp, mm). The skull specimens underwent EDTA decalcification, paraffin embedding, and sectioning, followed by hematoxylin‐eosin (H&E), Sirius red, and Masson's trichrome staining. Finally, immunofluorescence staining was performed to evaluate angiogenesis and osteogenic efficacy within the bone defect area.

### 2.11 Statistical Analysis

All the data are presented as the means ± standard deviations (SD). The number of biologically independent samples (n) for each experiment is indicated in the corresponding figure legends. GraphPad Prism 9.5.1, Adobe Photoshop 2022, and Microsoft PowerPoint 2024 were used for graphing and figure preparation. One‐way analysis of variance was used for statistical analysis. For data with equal variances, Dunnett's test or the least significant difference (LSD) test was used for multiple comparisons. For data with unequal variances, Dunnett's T3 test was applied. The data are denoted with the following symbols: (^*^) for a probability less than 0.05 (*p* < 0.05), (^**^) for *p* < 0.01, (^***^) for *p* < 0.005, and (^****^) for *p* < 0.0001.

## 3 Results and Discussion

### 3.1 Single‐Cell RNA Sequencing of Bone Tissue From Type 2 Diabetic Mice

**FIGURE 2:**
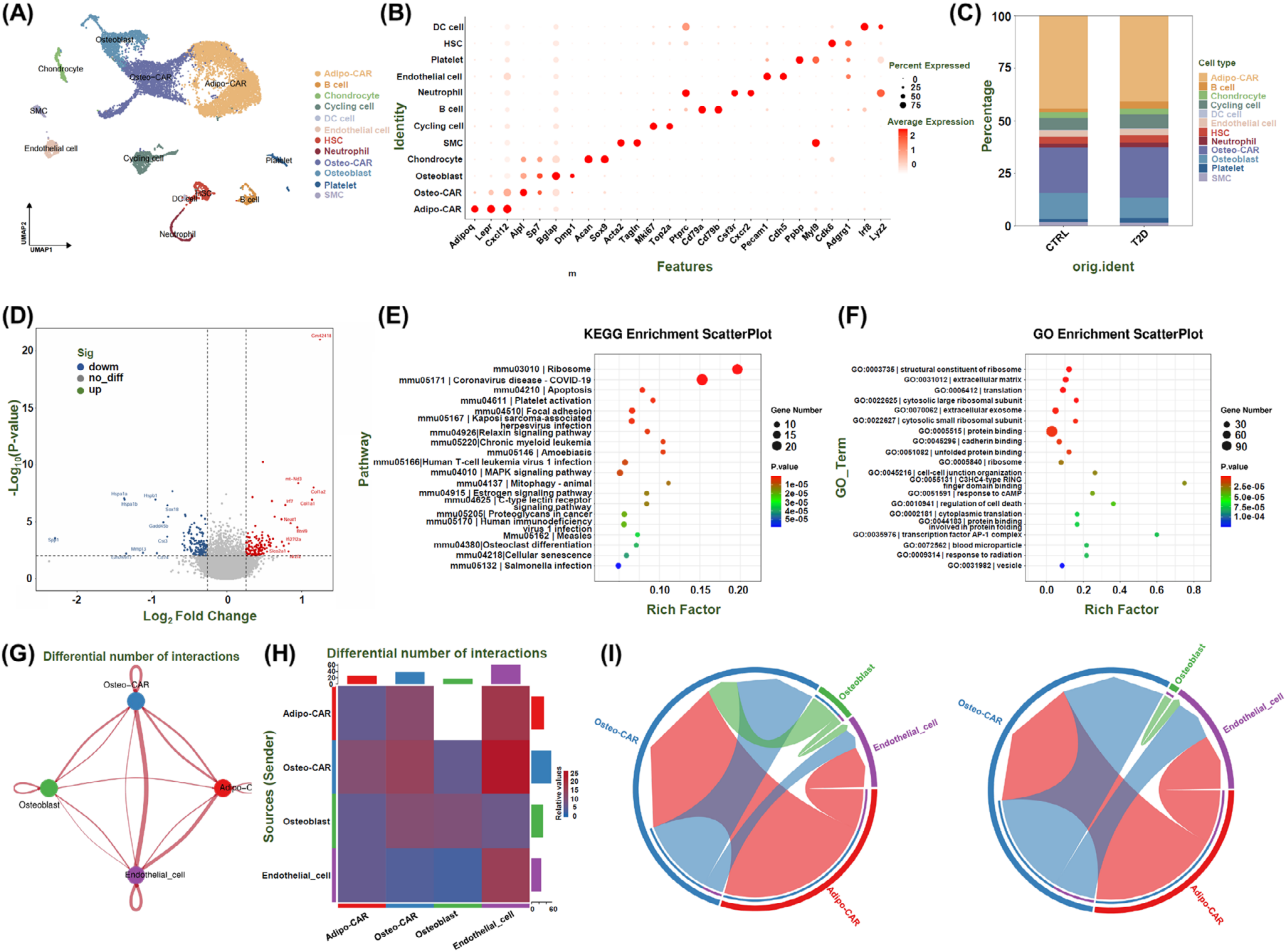
The single‐cell sequencing of bone tissue from the T2M and Control groups. (A) Cell types identified by uniform manifold approximation and projection (UMAP). (B) Bubble diagram showing marker genes in each cell subgroup. (C) Differences in cell composition between the T2M and Control groups. (D) The volcano plot showing the differential gene expression between the T2M and Control groups. (E) KEGG and (F) GO enrichment analysis of the differential genes. (G) Network plot showing ligand receptor interactions underlying the cross‐talk between osteoblastic and endothelial cells. (H) Heat map showing the interaction strength between the sender and receiver cell. (I) Noggin‐mediated cell‐to‐cell interaction (L: Control; R: T2M).

Public scRNA‐seq data (GEO: GSE221936) from T2M and control mice samples were integrated and analyzed to clarify the cellular heterogeneity in the bone microenvironment under T2M conditions. This analysis was intended to define disease‐relevant pathological features in a type 2 diabetic bone microenvironment, rather than to imply that all forms of diabetes share identical mechanisms. Using unsupervised clustering and expression of classical marker genes, 12 major cell types were identified (Figure 2A,B). Proportionality analysis revealed a tendency in the endothelial cell population in the T2M group to increase compared to the control (Figure 2C), suggesting a potential link between angiogenesis and osteogenesis in the T2M model. Analysis of endothelial cells indicated that the expression of genes, including CD31 and *Emcn* was downregulated, which may be consistent with the characteristics of H‐type vessels known for their osteo‐coupling function (Figure 2D). The expression of extracellular genes (*Vegf and Noggin*) in endothelial cells was substantially altered in the T2M group compared to the control. KEGG enrichment analysis indicated that the differentially expressed genes were substantially enriched in the MAPK signaling pathway and pathways related to osteogenic differentiation (Figure 2E). GO enrichment analysis indicated that the differentially expressed genes were substantially enriched in biological processes, including cAMP energy metabolism, oxidative energy production from organic compounds, and cell–cell junction organization (Figure 2F). Taken together, these data suggest that endothelial cells in the T2M group may exhibit a dysfunctional state, accompanied by metabolic reprogramming and altered angiogenic signaling, possibly associated with disturbed energy metabolism regulation.

Given this critical role in maintaining bone formation and energy metabolism homeostasis, an analysis of the communication between endothelial cells and osteoblasts. Alterations in the number of ligand–receptor pairs between endothelial cells and osteoblasts were evident in the T2M group (Figure 2G). Specifically, Noggin‐mediated interaction demonstrated marked differences in both the number of ligand–receptor pairs and communication strength (Figure 2H), which may indicate disruption of this regulatory axis under diabetic conditions. A single‐cell transcriptomic atlas of the bone microenvironment in T2M mice was constructed and indicated disease‐associated alterations in cellular composition and intercellular communication. Endothelial cells in the T2M group exhibited dysregulated expression of the characteristic markers, *CD31* and *Emcn*, which may reflect functional abnormalities related to H‐type vessel‐associated endothelial activity. Pathway enrichment analysis supported this interpretation, as the identified key signaling pathways constitute the core molecular basis regulating the formation and function of H‐type vessels. Cell communication analysis identified disrupted Noggin signaling from endothelial to osteoblastic cells as a key finding (Figure 2I), which may potentially link vascular dysfunction to impaired bone formation and provide important insights into the underlying mechanisms of diabetic bone disease.

Collectively, single‐cell analyses demonstrated that T2M may remodel the bone microenvironment, which is characterized by functional impairment of osteogenesis‐coupled H‐type vessels. This may, in turn disturb the communication network with osteoblasts, with its core mechanism being dysregulation of the Noggin signaling pathway [34]. These observations do not directly determine the architecture of the scaffold, but they provide a clear biological rationale for developing a regenerative material capable of restoring the local microenvironment required for H‐type vessel–associated repair. Based on this pathological insight, we reasoned that an effective scaffold for diabetic bone repair should not only mimic the structural characteristics of the periosteum but also provide electroactive and biofunctional cues to rescue endothelial dysfunction and favor H‐type vessel‐associated regeneration.

### 3.2 Preparation and Characterization of Janus Piezoelectric Scaffolds

**FIGURE 3:**
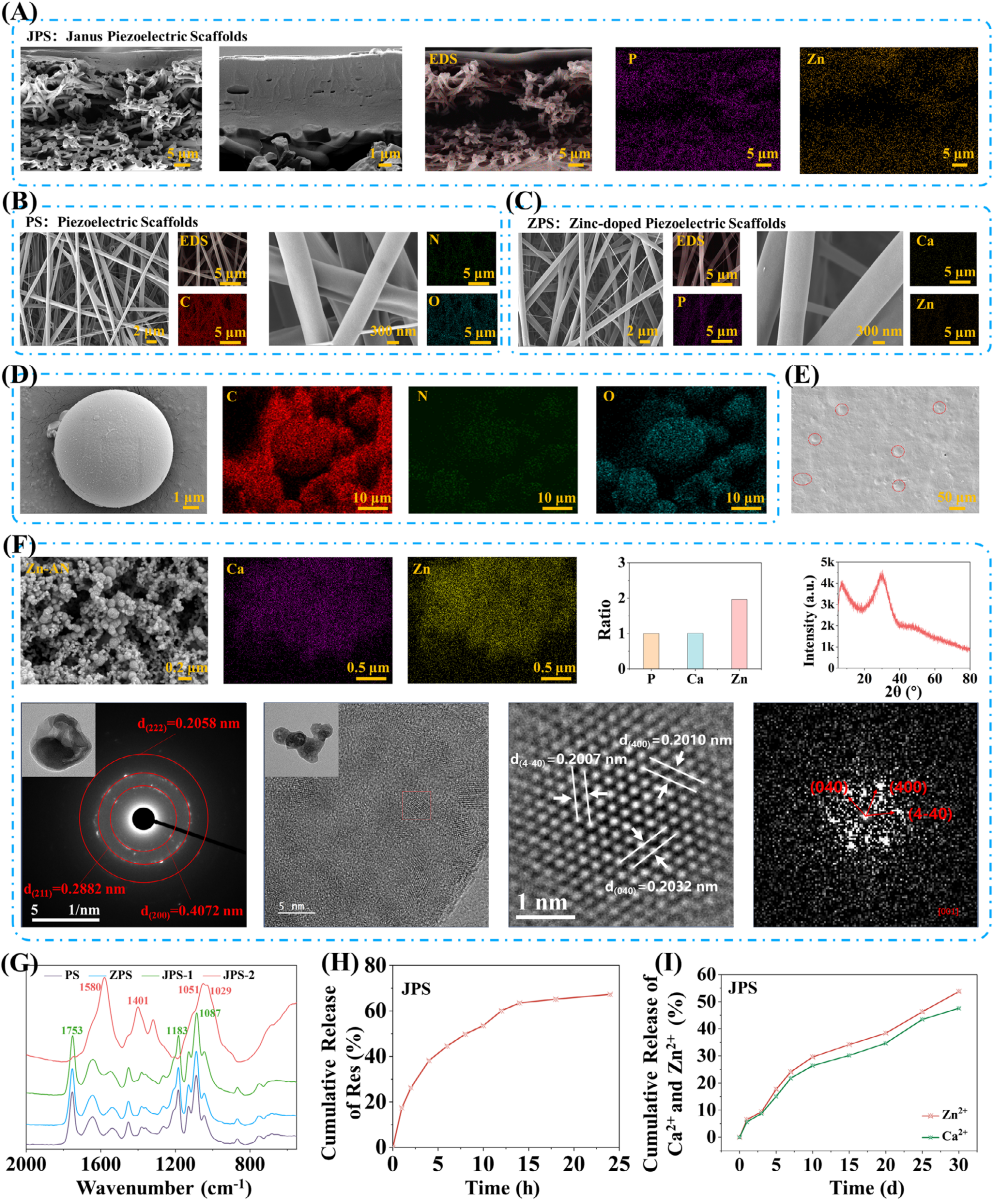
Preparation and characterization of Janus piezoelectric scaffolds. (A) Cross‐sectional morphology and element mapping of the bracket of the JPS. SEM images and EDS images of (B) PS, (C) ZPS, and (D) the resveratrol‐loaded carboxymethyl chitosan microspheres. (E) The surface morphology of the microsphere coating of JPS. (F) The SEM, EDS, element ratio statistics, XRD, and TEM of nano‐particle Zn‐AN. (G) FTIR spectra of all scaffolds. Cumulative release rate of (H) Res and (I) functional ion (Zn^2+^ and Ca^2+^).

Janus piezoelectric scaffolds integrate a gelatin–PLLA membrane fabricated via electrospinning and a resveratrol‐loaded carboxymethyl chitosan microspheres composite hydrogel (Figure S1). A genipin‐mediated chemical crosslinking strategy enables autonomous and robust integration of the two layers at the interface. Fibers with a three‐dimensional network structure were embedded within the composite hydrogel layer (Figure 3A). PS exhibited a three‐dimensional network structure with interwoven fiber alignment, resulting in structural stability and favorable spatial connectivity (Figure 3B). The zinc‐doped apatite nanoparticles effectively preserved this characteristic of the scaffold (Figure 3C). The characteristic elements, Ca and Zn, were detected in the ZPS scaffold.

The structure determines the function. Based on this, the constituent parts of JPS were characterized. The resveratrol‐loaded microspheres exhibited a near‐perfect spherical morphology (Figure 3D). The EDS analysis indicated the spherical aggregation of the elemental distribution. Spherical regions on the surface of the JPS microsphere coating (Figure 3E). Zinc‐doped apatite nanoparticles (Zn‐AN), which are crucial for osteogenesis and angiogenesis, exhibited a spherical particle morphology and contained abundant functional elements, such as Ca and Zn (Figure 3F). The mass ratios of P, Ca, and Zn are 1:1.01:1.96. The characteristic peak of hydroxyapatite was observed at 31.9°, according to Joint Committee on Powder Diffraction Standards card no. 09−0432. The maximum intensity peak of Zn‐AN appeared at 30.2°, which was caused by the lattice distortion induced by Zn incorporation. In TEM diffraction mode, a diffraction ring indicating the crystal structure was observed. The characteristic (211) plane of hydroxyapatite was identified with an interplanar spacing of 0.2882 nm. The high‐resolution crystal phase was analyzed. Lattice fringes with d‐spacings of 0.2010, 0.2007, and 0.2032 nm were observed, corresponding to the (400), (4‐40), and (040) crystal planes, respectively. This well‐defined crystal structure may have contributed to the enhanced piezoelectric performance of the material.

FTIR spectra indicated similar spectral patterns for PS, ZPS, and JPS‐1 (electrospinning fiber side), all displaying the characteristic peaks of the PLLA matrix, including peaks 1753, 1183, and 1087 cm^−^^1^ corresponding to functional groups C═O, C─O─C, and C─O, respectively. For JPS‐2 (gel side), the characteristic peaks of the carboxymethyl chitosan backbone were observed at 1051 and 1029 cm^−^^1^. Simultaneously, the characteristic peak at 1580 cm^−^^1^, which corresponds to the C═N bond, was observed, confirming the successful formation of the genipin‐crosslinked gel (Figure 3G).

The controlled release of active substances in the Janus piezoelectric scaffolds indicates their osteogenic potential. The sustained release of resveratrol from the microspheres persisted for 24 h, with a cumulative release of 67% (Figure 3H). The initial release of the drug facilitated the initiation of energy metabolism. The release of Ca and Zn continues for up to 30 d, which is attributed to the slight solubility of apatite in water. By day 30, the release rates of Zn and Ca reached 53.8% and 47.6%, respectively (Figure 3I). In summary, the synthesized Janus piezoelectric scaffolds exhibited physicochemical properties that demonstrated their osteogenic potential.

### 3.3 The Piezoelectric Properties of Janus Piezoelectric Scaffolds

**FIGURE 4:**
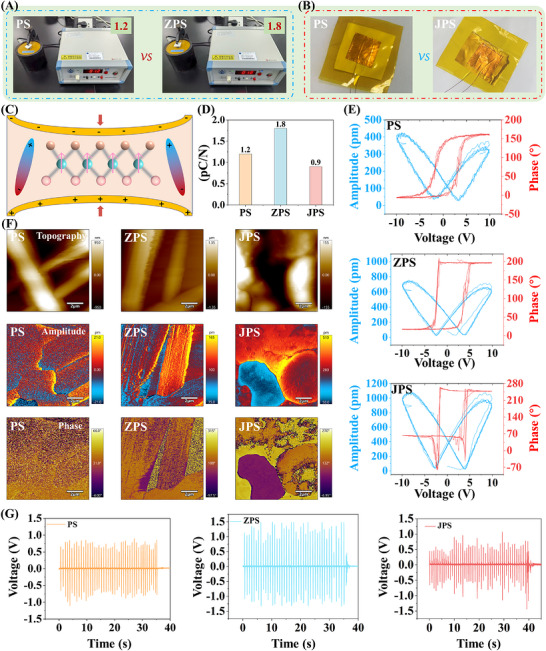
The piezoelectric properties of Janus piezoelectric scaffolds. Digital photo of (A) the piezoelectric constant (d_33_) testing, and (B) the encapsulated piezoelectric device. (C) Schematic diagram of the mechanism by which scaffolds generate the piezoelectric effect. (D) The d_33_ value of scaffolds. (E) PFM butterfly loop and hysteresis loop of scaffolds. (F) Topography, amplitude image, and phase image of scaffolds. (G) The output voltage signal strength of the device over 35 s.

The digital photographs indicate the piezoelectric performance testing process of the scaffold (Figure 4A and Figure S2) and the encapsulated piezoelectric device (Figure 4B and Figure S3). The PLLA molecular chains inherently contain polar groups, primarily the carbonyl groups (C═O). When subjected to mechanical forces, dipole moments, such as carbonyl groups on the molecular chains, undergo preferential orientation in specific directions. To compensate for this polarization change, the material surface generates an equivalent amount of induced charge, thereby producing a voltage (Figure 4C). The incorporation of zinc‐doped apatite nanoparticles enhanced the piezoelectric constant of ZPS (1.8 pC/N) by 1.5‐fold compared to PS (1.2 pC/N; Figure 4D). With the introduction of a microsphere coating on the surface, the piezoelectric constant of JPS was 0.9 pC/N. This value is in close agreement with the reported piezoelectric constant of cranial bone (∼1 pC/N) [35].

PFM was used to characterize the microscale piezoelectric properties of the scaffolds. The topography image clearly indicates the individual fibers of PS and ZPS, as well as the microsphere–gel morphology of JPS (Figure 4F), which is consistent with the SEM and TEM observations. All three scaffolds exhibited well‐defined contours in their amplitude images. The maximum amplitudes for PS, ZPS, and JPS were 422, 743, and 1069 nm, respectively (Figure 4E). The amplitude increases in JPS originated from the weak piezoelectric materials (carboxymethyl chitosan). Based on the phase image results, the ZPS exhibited the most ideal rectangular hysteresis loop. This confirms that the incorporation of Zn‐AN enhances piezoelectric performance, whereas the introduction of the microsphere coating leads to a reduction in piezoelectricity, consistent with the piezoelectric constants d_33_ results. The responsiveness of the scaffolds to mechanical forces was validated by simulating the output voltage. When a 2N steady‐state or dynamic force was applied to the device surface (Figure 4G), the output voltages generated by PS, ZPS, and JPS were 0.7, 1.2, and 0.6 V, respectively. Therefore, Janus piezoelectric scaffolds could generate electrical signals in response to mechanical stimulation, indicating their potential piezoelectric responsiveness in vivo, and regulate cellular behavior. However, the local defect‐site mechanical environment and the corresponding in vivo electrical output were not directly quantified in the present study.

### 3.4 Evaluation of Biocompatibility

**FIGURE 5:**
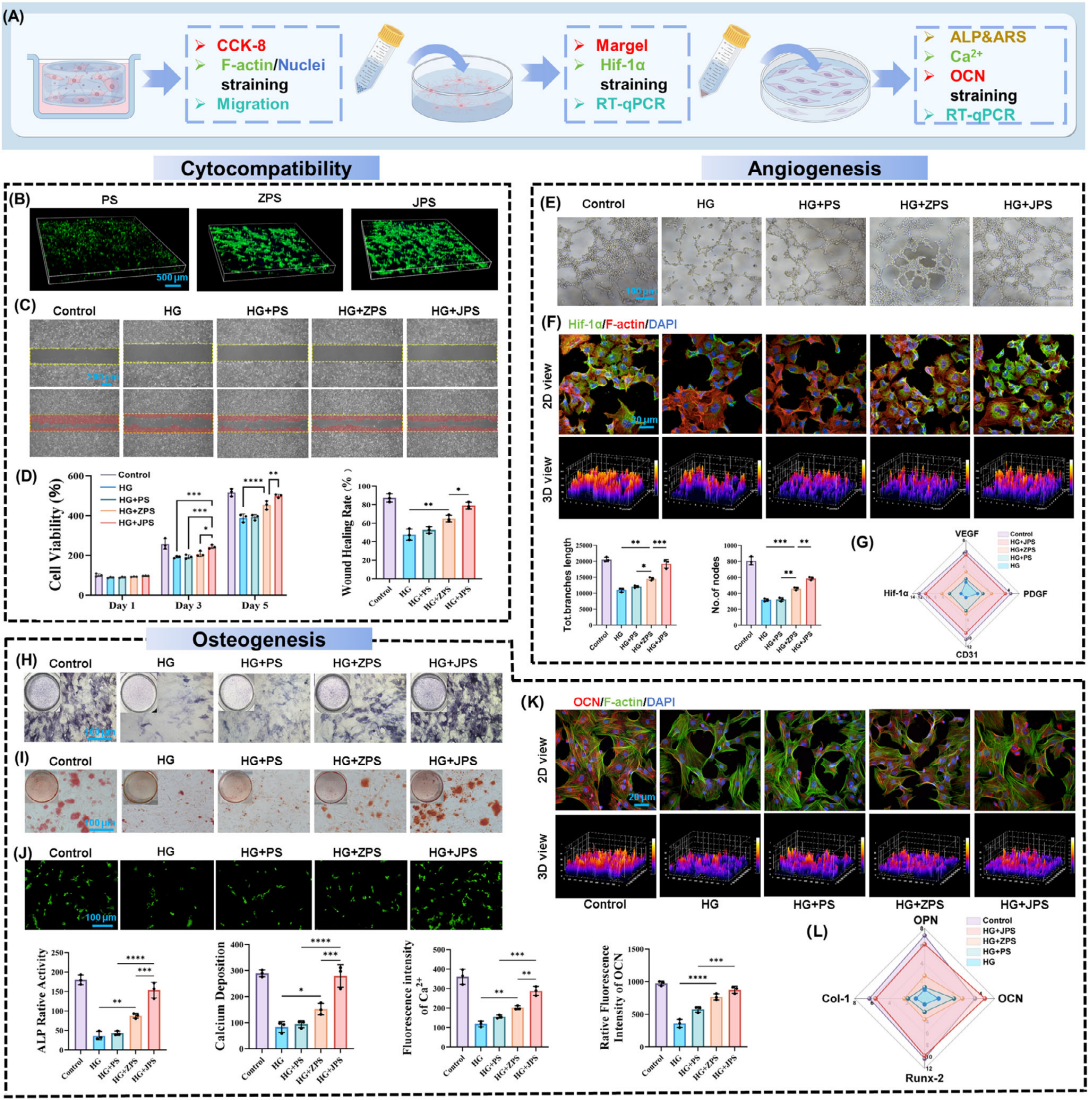
In vitro osteogenesis and angiogenesis effects of different treatments. Control, normal‐glucose group (5.5 mm glucose); HG, high‐glucose group (25 mm glucose) without scaffold treatment; PS, ZPS, and JPS denote the corresponding scaffold‐treated groups under high‐glucose conditions. (A) Schematic illustration of in vitro cell tests. (B) The cytoskeletal F‐actin (green) distribution of BMSCs cultured under different treatments for 3 d. The cell nuclei were counterstained with DAPI (blue) (*n* = 3 per group). (C) Scratch tests of the BMSCs under different treatments for 48 h (*n* = 3 per group). (D) Quantification of the cell proliferation and distance of the scratch area (*n* = 3 per group). (E) Matrigel tube‐forming assays of the HUVECs under different treatments using conditioned medium collected after 24 h of treatment and quantified after 6 h of tube formation (*n* = 3 per group). (F) IF images of HUVECs stained by the HIF‐1α (green), phalloidine (red), and DAPI (blue) under different treatments for 48 h (*n* = 3 per group). (G) Relative mRNA expression of angiogenesis‐related genes (*Hif‐1α, CD31, PDGF, VEGF*) (*n* = 3 per group). (H) ALP protein staining at day 14 (*n* = 3 per group). (I) ARS protein staining at day 21 (*n* = 3 per group). (J) Fluorescence plot of intracellular calcium ions (*n* = 3 per group). (K) IF images of BMSCs stained using OCN (red), phalloidine (green), and DAPI (blue) under different treatments (*n* = 3 per group). (L) Relative mRNA expression of osteogenesis‐related genes (*Col‐1, Runx‐2, OPN, OCN*) (*n* = 3 per group). Data are presented as mean ± SD, ^*^ *p* < 0.05, ^**^ *p* < 0.01, ^***^ *p* < 0.001 and ^****^ *p* < 0.0001. Statistical analysis was performed as follows: quantitative data associated with panels C–E, G–J, and L were analyzed using one‐way ANOVA followed by Dunnett's post hoc test for planned comparisons of scaffold‐treated groups vs. the HG group; additional pairwise comparisons indicated by horizontal significance bars were analyzed using the LSD post hoc test.

Figure 5A illustrates the experimental procedure used for biocompatibility testing. Cells in both the high glucose (HG) and PS groups demonstrated reduced viability and shrunken morphology in cytoskeleton‐staining images (Figure 5B). Compared to the PS group, the ZPS group demonstrated uniformly distributed cells with improved spreading, typically exhibiting spindle‐like or polygonal morphologies. The JPS group showed tight cell adhesion and full spreading on the Janus scaffold surface, with clearly extended pseudopodia. These observations may indicate good cytocompatibility of the scaffolds and suggest acceptable biosafety under the present experimental conditions. The HG and PS groups showed the lowest cell migration rates in the scratch assay, with no statistical differences between them (Figure 5C). The ZPS group demonstrated a higher migration rate than the HG and PS groups (*p* < 0.05). The scratches in the JPS group healed almost completely, similar to the results observed in the control group. These findings suggest that the JPS scaffolds may promote the migration of BMSCs under high‐glucose conditions, which may be beneficial for accelerating the repair process of diabetic bone defects.

The OD values of all groups increased with prolonged culture time (Figure 5D). The OD values in the various groups were similar on day 1 (*p* > 0.05), indicating the absence of acute toxicity during the initial phase. From day 3 onward, differences in OD values were evident in the groups; the high glucose (HG) group had lower values than the control group (*p* < 0.01), demonstrating that the high‐glucose environment markedly inhibited cell proliferation. The ZPS group had higher OD values than the PS and HG groups (*p* < 0.05), indicating that Zn^2+^ release partially alleviated high glucose‐induced toxicity. The JPS group showed the highest OD values of all the groups (*p* < 0.01), suggesting that the combined action of the piezoelectric effect and resveratrol release may have partially reversed the high glucose‐induced suppression of cell proliferation.

### 3.5 Evaluation of In Vitro Angiogenesis Capability

In this section, HUVECs were used as a standard endothelial model to evaluate the general pro‐angiogenic effects of the scaffolds under high‐glucose conditions, rather than to directly define bone‐specific type H vessel identity. The tube formation assay indicated complete vascular networks in the control group. The HG and PS groups only formed incomplete and fragmented tubular structures, exhibiting reduced total length, fewer branch points, and a lower number of meshes (Figure 5E). The ZPS group demonstrated higher vascular formation parameters than HG and PS groups (*p* < 0.05), which may indicate that Zn doping confers a certain pro‐angiogenic potential to the scaffolds. The JPS group exhibited more pronounced pro‐angiogenic effects than other groups. The total tubule length (1876 ± 132 µm), and number of branch points (562 ± 21) in the JPS group were higher than those in the scaffold‐treated group (*p* < 0.01). These findings suggest that the synergistic action of the piezoelectric effect, Zn^2^^+^ release, and resveratrol release may enhance the tube‐forming ability of HUVECs under high‐glucose conditions.

At the molecular level, mRNA expression patterns of *VEGF*, *Hif‐1α*, and *CD31* were consistent with the tube formation assay results based on qPCR analysis (Figure 5G). Compared to the control and HG groups, the mRNA expression levels of *VEGF*, *Hif‐1α*, and *CD31* in the ZPS group were all up‐regulated to some extent, which may be consistent with the known biological effects of Zn^2+^. The JPS group exhibited the most significant upregulation. As an upstream regulator of the hypoxic response and VEGF pathway, the most pronounced change in Hif‐1α expression may suggest that the piezoelectric field and the released bioactive molecules mimic a hypoxia‐like, pro‐angiogenic signal. *Hif‐1α*, an endothelial cell marker and adhesion molecule, was substantially upregulated, corroborating the tube formation results and indicating full activation of endothelial cell function. In the JPS group, the fluorescence intensity of *Hif‐1α* was markedly enhanced, with a continuous, well‐defined linear distribution, outlining intercellular junctions and lumen‐like structures formed between endothelial cells (Figure 5F). This pattern appeared to correspond to the mature tubular structures observed in the tube formation assay.

The scRNA‐seq analysis in Figure 2 suggested impaired endothelial–osteoblastic communication in the diabetic bone microenvironment. In contrast, the in vitro assays in Figure 5 were not intended as a direct functional validation of this interaction, but rather as parallel evidence showing that JPS improves both angiogenic and osteogenic phenotypes under high‐glucose conditions. The synergistic effect of piezoelectricity and drug release may be one of the key factors contributing to the superior performance of the JPS group compared to the other groups, at the level of general endothelial angiogenic behavior. We speculate that the mechanical activities of the cells, including migration and contraction, may generate subtle stresses on the scaffolds. These stresses may be captured by the piezoelectric component of the scaffold and converted into a localized micro‐electric field [36], while this process may also be accompanied by simultaneous drug release [37]. Together, these effects may have contributed to the accelerated migration, interconnection, and maturation of HUVECs into stable three‐dimensional tubular networks, thereby potentially enhancing the pro‐angiogenic response under high‐glucose conditions.

### 3.6 Evaluation of In Vitro Osteogenic Capability

The JPS group exhibited ALP activity second only to the control group and was higher than that of the other experimental groups (*p* < 0.05; Figure 5H) after 14 d of culture. Compared to the other intervention groups, the JPS group exhibited the most obvious blue‐purple staining, the largest precipitate area, and the most densely distributed deposition. After 21 d of culture, both HG and PS groups showed minimal amounts of red mineralized nodules (Figure 5I). The ZPS group demonstrated an increase in the number and size of nodules. JPS exhibited the most marked mineralization effect, forming a large number of dense and extensive red calcium nodules, similar to those in the control group. The quantitative absorbance value of JPS was significantly higher than that of the other interventions (*p* < 0.05), which may indicate that it had the strongest ability to promote matrix mineralization under high glucose conditions. The fluorescence plot of intracellular calcium ions indicated a similar response (Figure 5J).

For both early (*Runx‐2* and *COL‐1*) and late markers (*OPN* and *OCN*), the JPS group demonstrated the most substantial upregulation in gene expression (*p* < 0.05; Figure 5L). This result was generally consistent with the ALP and ARS staining, suggesting that the scaffolds may promote osteogenic differentiation in a coordinated manner. Compared to the HG and PS groups, the ZPS group exhibited an enhanced OCN fluorescence signal (red fluorescence) (Figure 5K). The JPS group showed the strongest OCN fluorescence intensity overall, whereas the ZPS group also demonstrated a visibly enhanced signal relative to the HG and PS groups, with fluorescence features that may be associated with increased extracellular matrix deposition.

### 3.7 Evaluation of In Vitro Vascular Organogenesis Capability

**FIGURE 6:**
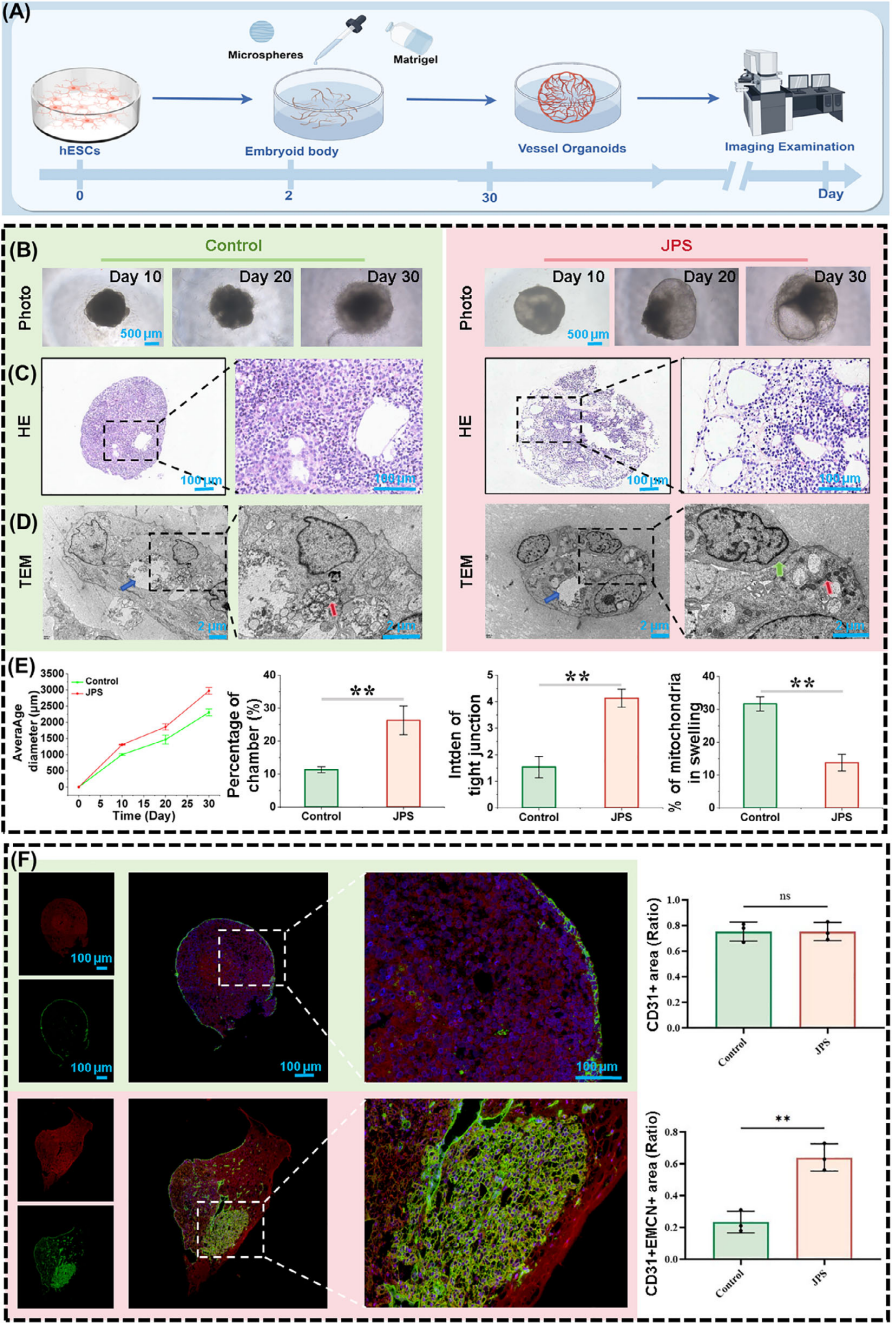
Janus piezoelectric scaffolds used to construct vessel organoids in vitro. (A) Schematic diagram of the procedure used to generate vessel organoids. (B) Morphology of organoids on days 10, 20, and 30 (*n* = 3 per group). (C) HE staining of organoids (*n* = 3 per group). (D) TEM image of organoids. Tight junction (green arrow); mitochondria (red arrow); lumen (blue arrow) (*n* = 3 per group). (E) Quantification of the growth states of organoids from each group (*n* = 3 per group). (F) IF images of organoids stained by the EMCN (green), CD31 (red), and DAPI (blue) under different treatments (*n* = 3 per group). Data are presented as mean ± SD, ^*^ *p* < 0.05, ^**^ *p* < 0.01, ^***^ *p* < 0.001 and ^****^ *p* < 0.0001. Statistical analysis was performed as follows: quantitative data associated with panels B–F were analyzed using an unpaired two‐tailed Student's *t*‐test.

Incorporating vascularized organoids into the osteogenic process represents not only a critical breakthrough in biomimetic design but also a pivotal step toward functional bone‐regeneration therapy (Figure 6A). To evaluate the effect of JPS on vascular organoid formation, optical microscopy morphological observations were conducted on days 10, 20, and 30. On day 10, cell aggregation and initial formation of cytoplasmic cord‐like structures were observed in the control and JPS groups (Figure 6B). On day 20, the JPS group developed highly intricate and dense tubular network structures, suggesting features of a more mature vascular network. On day 30, the vascular network in the JPS group reached maturity, with the main vessels and branches constituting a well‐defined, dense circulatory system‐like architecture. The diameters of the control and JPS groups were 3580 ± 251 and 2486 ± 232 µm, respectively (*p* < 0.01).

H&E staining results (Figure 6C) were consistent with the optical microscopy. Sections from the JPS group displayed vascular‐like structures with complete and clearly defined lumens, and the endothelial cells were neatly arranged along the lumen in a flattened morphology. Moreover, spindle‐shaped pericytes, or smooth muscle cells, adhered to the periphery of vascular‐like structures, which may indicate a more advanced stage of vascular maturation and stabilization. Observations in the control group indicated vascular‐like structures exhibiting heterogeneous lumen sizes, as well as partially collapsed or incomplete lumens. Endothelial cells were loosely arranged, and mature vascular structures with pericyte attachments were rarely observed. Quantitative analysis indicated that the proportion of functional capillary‐like compartments in the JPS group was 31 ± 5%, which was higher than the 12 ± 6% observed in the control group (Figure 6E, *p* < 0.01).

TEM analysis was conducted to assess the maturity and functional characteristics of the vascular organoids at the subcellular level (Figure 6D). The control group exhibited poor vascular structural maturity. The JPS group demonstrated highly mature, vascular‐like structures characterized by well‐developed adhesive junctions between endothelial cells, which were greater than those in the control group (4.2 ± 0.5 units per µm vs. 1.8 ± 0.3 units per µm, *p* < 0.01). The swelling rates of the JPS and control groups were 12 ± 6% and 31 ± 5%, respectively (*p* < 0.01), which may reflect a more preserved ultrastructural state in the JPS group (Figure 6E). Numerous fenestrated structures covered by a single‐layer diaphragm were observed in the capillary‐like structures of the JPS group. These features may resemble ultrastructural characteristics reported for H‐type vessels in vivo (red arrows) [38].

Immunofluorescence co‐staining for CD31 (a pan‐endothelial marker) and EMCN (a marker associated with type H vessel‐like endothelial features in bone) was used to characterize the vascular generation status. The control and JPS groups expressed CD31, which was consistent with the observations from optical microscopy and H&E staining. However, the control group exhibited extremely weak and fragmented EMCN signals with indistinct co‐localization with CD31, indicating immature vascular structures. In the JPS group, extensive and distinct co‐localization of EMCN with CD31^+^ signals was observed, with the proportion of double‐positive vessels reaching 53.3 ± 4.2%, which was higher than that in the control group (*p* < 0.001; Figure 6F). These findings suggest that JPS may favor the formation of vascular features associated with a type H vessel‐like phenotype, particularly in the main trunks and primary branches of the vascular network, which showed denser distribution and more continuous signaling.

### 3.8 Mechanistic Analysis of the Pro‐Angiogenic Effect of JPS

**FIGURE 7:**
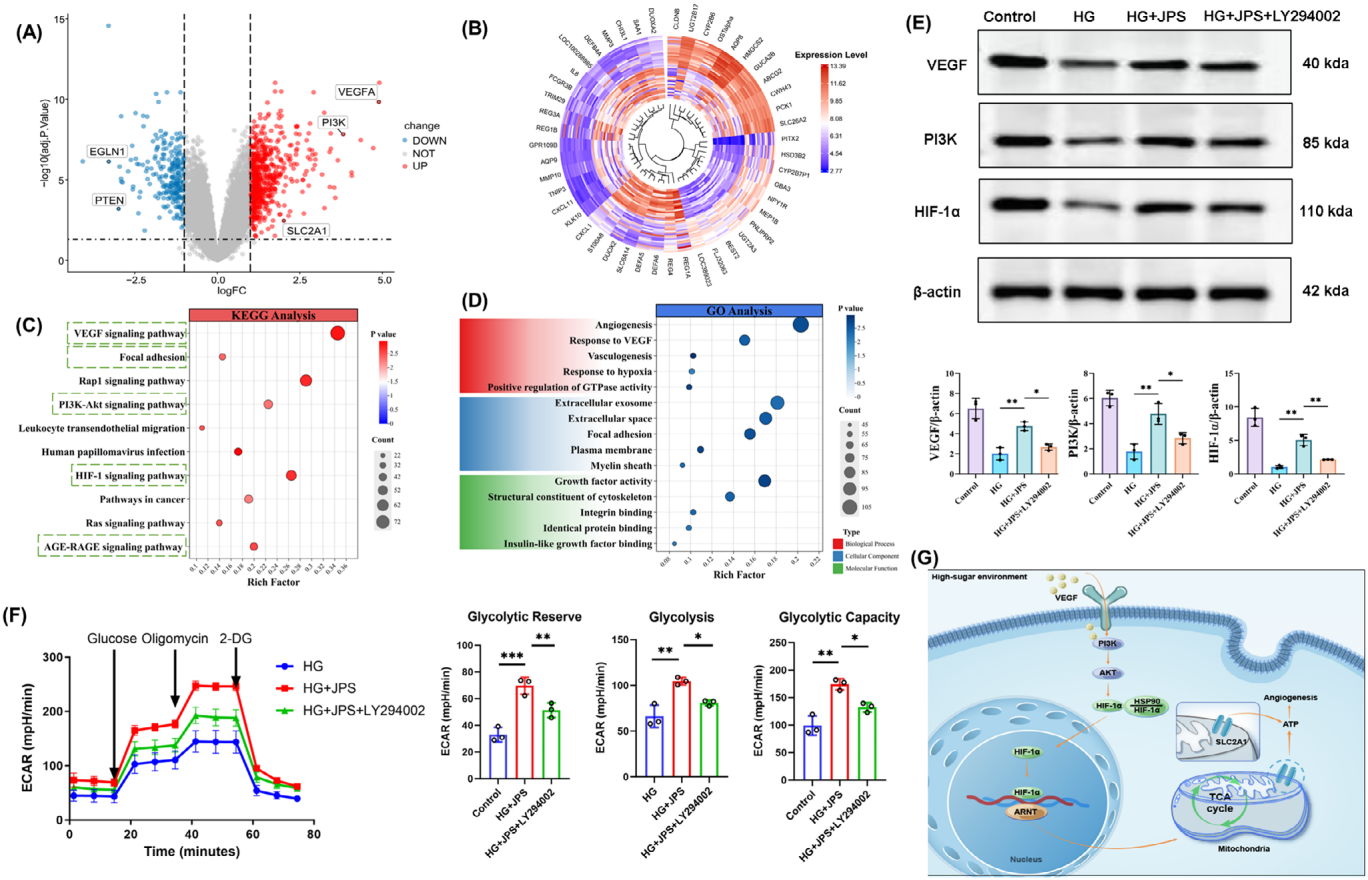
Transcriptome sequencing data validation. (A) Volcano diagram of the differential expression of RNA in the HG and JPS groups. (B) Heat map depicting gene expression changes in the HG and JPS groups. (C) KEGG enrichment results. (D) GO enrichment results. (E) Western blotting showing the relative expression levels of VEGFA, PI3K, and HIF‐1α between the different groups (*n* = 3 per group). (F) Seahorse glycolysis analysis of HUVECs under different treatment conditions (*n* = 3 per group). (G) Schematic of the proposed mechanism underlying the pro‐angiogenic effect of JPS under high‐glucose conditions. Data are presented as mean ± SD, ^*^ *p* < 0.05, ^**^ *p* < 0.01, ^***^ *p* < 0.001 and ^****^ *p* < 0.0001. Statistical analysis was performed as follows: quantitative data in panels E and F were analyzed using one‐way ANOVA followed by the LSD post hoc test.

RNA‐seq was conducted on the HG and HG+JPS groups to investigate the proangiogenic mechanisms of the JPS piezoelectric effect combined with resveratrol release. Analysis of differentially expressed genes (DEG) indicated that 432 genes were upregulated and 321 genes were downregulated in the JPS group. The volcano plot displays the distribution of DEGs (Figure 7A); the heatmap further indicates distinct gene expression patterns between the groups (Figure 7B). KEGG enrichment analysis indicated that the pathways most relevant to angiogenesis (VEGF, PI3K‐Akt, and HIF‐1 signaling pathways) were among the most significantly enriched pathways in the JPS group (Figure 7C). Moreover, cellular adhesion and migration processes were also profoundly regulated, as suggested by the significant enrichment of the Focal Adhesion pathway.

The results of GO enrichment analysis (Figure 7D) indicated that, in biological processes (BP), the differential terms were angiogenesis, response to VEGF, and vasculogenesis. For cellular components (CC), the significant terms included extracellular exosomes and focal adhesions. In the molecular function (MF) category, the enriched terms included growth factor activity and integrin binding. These changes are primarily associated with angiogenesis, adhesion, and migration. These results are consistent with the findings from the KEGG analysis, providing transcriptomic support suggesting that the JPS group may exhibit enhanced angiogenesis‐related signaling and endothelial functional activity under high‐glucose conditions.

To further elucidate the biological mechanisms underlying the pro‐angiogenic capacity of JPS, we performed western blotting in the Control, HG, HG+JPS, and HG+JPS+LY294002 groups to examine the expression of key proteins in angiogenesis‐related signaling pathways, including VEGFA, PI3K, and HIF‐1α. Compared with the HG group, the HG+JPS group showed increased protein expression levels of VEGFA, PI3K, and HIF‐1α (Figure 7E). However, these increases were markedly attenuated after treatment with LY294002, which may indicate that the pro‐angiogenic effect of JPS is at least partly associated with PI3K‐related signaling. In addition, Seahorse glycolysis analysis was performed to further evaluate the metabolic phenotype of endothelial cells under high‐glucose conditions. Compared with the HG group, the HG+JPS group exhibited a more active glycolytic profile, suggesting that JPS could partially modulate high glucose‐impaired endothelial metabolic reprogramming (Figure 7F). Compared with the HG+JPS group, the HG+JPS+LY294002 group showed a reduced glycolytic profile, suggesting that inhibition of PI3K may partially weaken the metabolic regulatory effect of JPS.

These findings suggest that JPS may promote endothelial angiogenic activity under high‐glucose conditions through coordinated regulation of angiogenesis‐related signaling and glycolytic metabolism. Specifically, the upregulation of VEGFA, PI3K, and HIF‐1α may suggest that JPS enhances angiogenesis‐related signaling pathways closely associated with endothelial sprouting and vascular remodeling. In addition, the enrichment of focal adhesion‐related pathways may indicate that the piezoelectric microenvironment and scaffold surface characteristics may facilitate endothelial adhesion, migration, and vascular network formation [39, 40]. Furthermore, the Seahorse glycolysis results suggest that JPS may partially remodel the metabolic phenotype of endothelial cells under diabetic‐like stress, which may provide functional metabolic support for angiogenic behavior.

In summary, our transcriptomic, protein‐level, and Seahorse data suggest that the pro‐angiogenic function of JPS may involve the coordinated regulation of angiogenesis‐related signaling and endothelial metabolic reprogramming (Figure 7G). Driven by the synergistic effects of piezoelectric stimulation, Janus scaffold architecture, and resveratrol release, JPS may enhance VEGFA‐, PI3K‐, and HIF‐1‐related pathways while improving the glycolytic phenotype of endothelial cells, thereby potentially supporting angiogenic responses under high‐glucose conditions. The attenuation of both protein expression and glycolytic activity by LY294002 further supports the important role of the VEGFA/PI3K/HIF‐1α‐related pathway in mediating the pro‐angiogenic activity of JPS.

### 3.9 In Vivo Bone Regeneration Potential of Janus Piezoelectric Scaffolds

**FIGURE 8:**
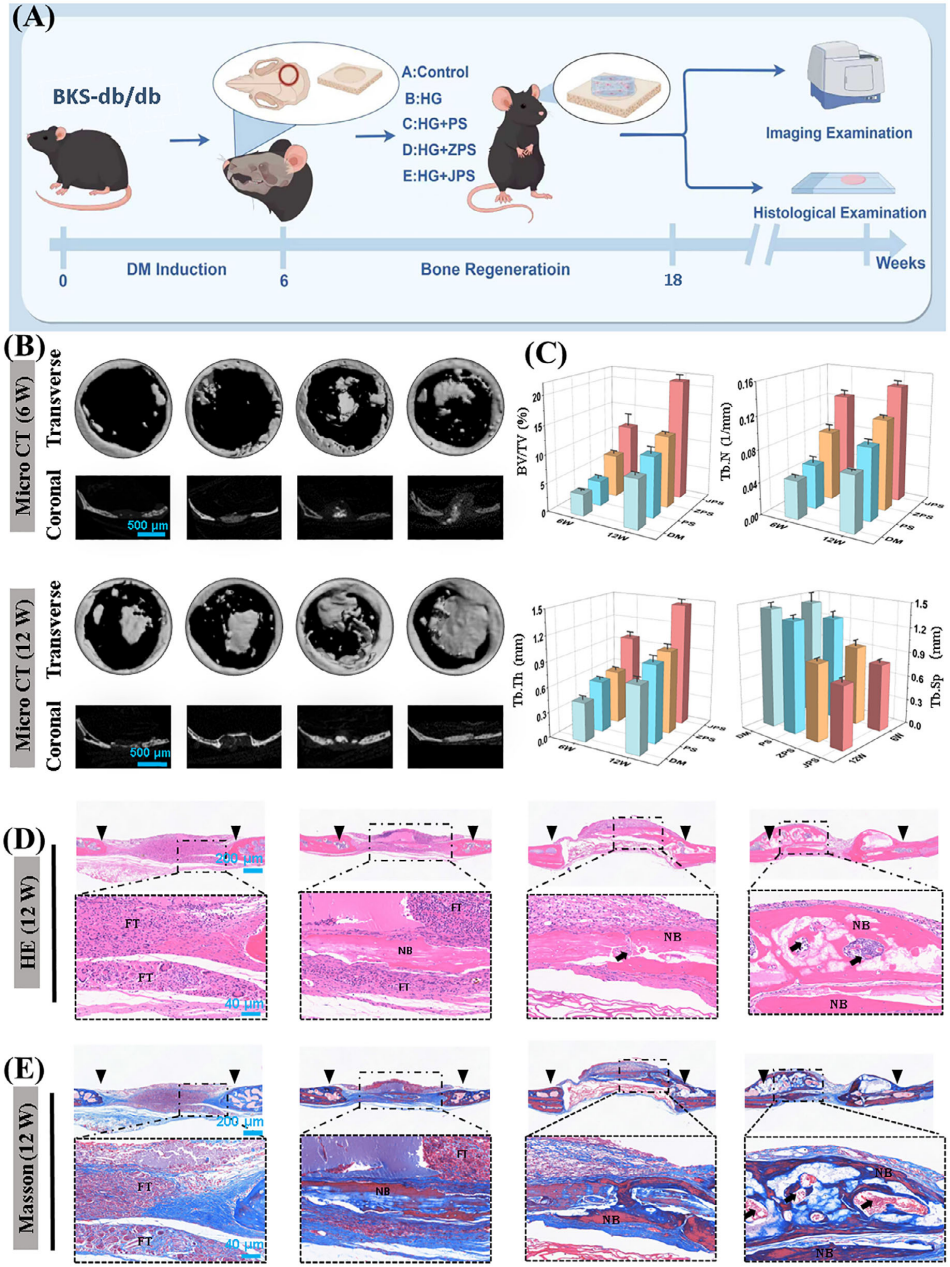
Evaluation of in vivo bone defect repair following scaffold implantation through micro‐CT and histological staining. (A) A schematic illustration of animal experiments. (B) Representative micro‐CT results of mice calvariae at 6− and 12‐w post‐operation (*n* = 5 per group). (C) Calculations of BV/TV, Tb.N, Tb.Th, and Tb.Sp of new bone in the calvarial defects (*n* = 5 per group). (D) H&E and (E) Masson's trichrome staining of histological sections at 12‐w post‐operation. FT = fibrous tissue; NB = newly‐formed bone; Black arrows = blood vessels (*n* = 5 per group). Data are presented as mean ± SD, ^*^ *p* < 0.05, ^**^ *p* < 0.01, ^***^ *p* < 0.001 and ^****^ *p* < 0.0001. Statistical analysis was performed as follows: quantitative micro‐CT data in panel C were analyzed using one‐way ANOVA followed by Dunnett's post hoc test, with the DM/Control defect group used as the reference group.

A critically‐sized (3 mm) cranial defect model in genetically diabetic db mice was established to evaluate the in vivo osteogenic performance of the Janus piezoelectric scaffolds (Figure 8A). In the micro‐CT images taken at week 6 (Figure 8B), the defect area in the HG group was primarily composed of low‐density soft tissue shadows with only a small number of newborn bone scabs. The PS and ZPS groups showed partial bridging with sparse trabecular structures, whereas the JPS group exhibited more new bone formation than the other groups and developed an initial bone network structure. The JPS group had a higher BV/TV (11.5%) than the other three groups, while demonstrating the lowest Tb.Sp (0.81 mm; Figure 8C), which may suggest an enhanced early osteogenic response.

In the micro‐CT images at week 12, the calvarial defect area in the JPS group was largely filled with abundant, newly‐formed, hypoechoic, mineralized tissue, making the defect boundaries almost indistinguishable. However, the defect area in the HG group remained clearly visible, with only minimal sparse bone tissue growing from the edges toward the center; the central region was predominantly filled with low‐density fibrous soft tissue. Only marginal callus formation was observed in the PS‐treated group. A similar pattern was observed in the PS group. The bone defect in the ZPS group was largely closed; however, the bone density was uneven, and minor unrepaired areas remain visible in the center of the defect. The JPS group demonstrated enhancement of all osteogenic indicators (*p* < 0.01). Compared to the BV/TV (8.5%) and Tb.Sp (1.43 mm) in the diabetic mice (DM) group, the JPS group exhibited BV/TV and Tb.Sp values of 21.1% and 0.75 mm, respectively (Figure 8C), which may indicate more effective bone defect repair under diabetic conditions.

H&E staining indicated that the HG and PS groups were predominantly composed of abundant fibrous connective tissue, with small amounts of irregular bone tissue (Figure 8D). The ZPS group exhibited more new bone matrix; however, its maturity was slightly lower. In the JPS group, abundant mature new bone trabeculae were observed, accompanied by complete bone marrow cavities and uniform cell distribution. Masson's trichrome staining indicated that the defect area in the JPS group exhibited extensive red‐stained new bone tissue, interwoven with a network of blue collagen fibers (Figure 8E), suggesting a more advanced stage of matrix deposition and tissue remodeling.

**FIGURE 9:**
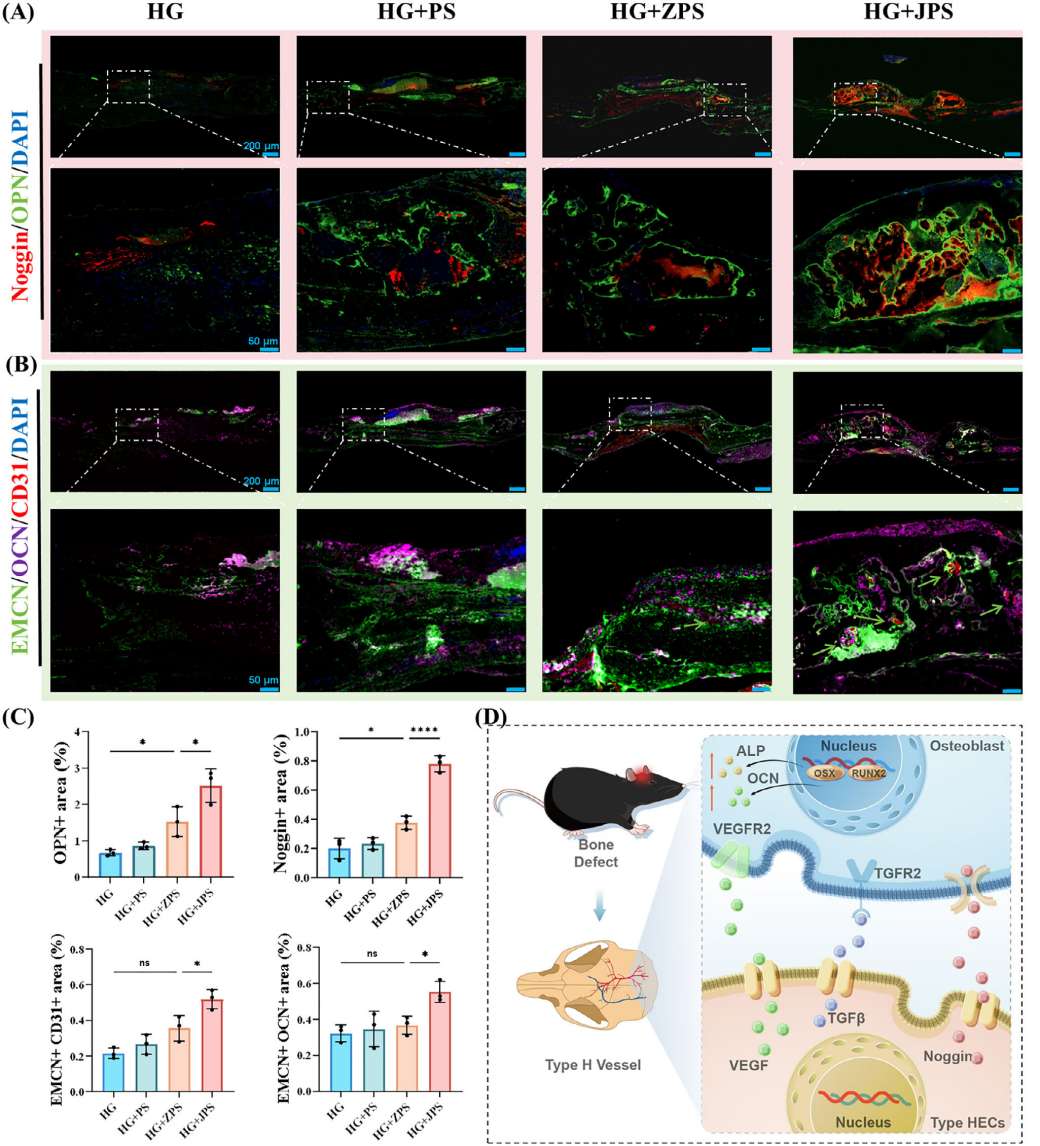
Co‐staining of osteogenic and angiogenic factors in bone defect repair following scaffold implantation using immunofluorescence staining. (A) Double Immunofluorescence staining of OPN (Green) and Noggin (Red); the nucleus is stained blue (*n* = 3 per group). (B) Triple immunofluorescence staining of OCN (Purple), EMCN (Green), and CD31 (Red); the nucleus is stained blue (*n* = 3 per group). (C) Semi‐quantitative results of osteogenic‐related proteins and H‐type vessel‐related proteins (*n* = 3 per group). (D) A schematic diagram of the process of promoting bone regeneration in the microenvironment of the cranial defect area through the stimulation of type H angiogenesis within the body. Data are presented as mean ± SD, ^*^ *p* < 0.05, ^**^ *p* < 0.01, ^***^ *p* < 0.001 and ^****^ *p* < 0.0001. Statistical analysis was performed as follows: quantitative immunofluorescence data in panel C were analyzed using one‐way ANOVA followed by Dunnett's post hoc test, with the DM/Control defect group used as the reference group.

Compared to single staining, immunofluorescence co‐staining analysis may provide a more comprehensive understanding of the close correlation between angiogenesis and osteogenic activity. To visually validate the proposed paracrine mechanism within bone‐vascular coupling, double immunofluorescence staining was conducted for Noggin, a key osteogenic factor that has been reported to be associated with H‐type vessel endothelium, and osteopontin (OPN), a marker for mature osteoblasts and nascent bone matrix. Strong Noggin immunoreactivity in the JPS group was localized near the CD31‐positive H‐type vasculature (Figure 9A). In areas of active bone regeneration, a significant proportion of OPN‐positive cells were encircled by or in direct contact with Noggin‐rich signals, which may suggest a paracrine regulatory relationship.

The JPS group exhibited dense CD31^+^/EMCN^+^ H‐type vascular structures within the new bone and surrounding tissues, with their quantity being substantially greater than that in other groups (Figure 9B,C). OCN signals closely surrounded the red H‐type vessels, where bone trabeculae were interwoven with the H‐type vascular network and extended jointly toward the defect center, collectively forming vascular–osteogenic unit structures. In contrast, the control groups displayed sparse vascular structures with weak co‐localization of osteogenic markers.

The immunofluorescence results may provide supportive visual evidence, at the morphological and spatial levels, of the bone–vascular coupling mechanism proposed in this study. The findings are generally consistent with those of an established bone repair model (Figure 9D). H‐type vessels created a specialized microenvironment for bone regeneration, not only supplying blood and nutrients to active osteogenic processes but also releasing various osteogenic factors (VEGF, TGF‐β, and Noggin) through paracrine signaling from their endothelial cells, directly stimulating and guiding the differentiation, maturation, and matrix secretion of adjacent osteoprogenitor cells.

In summary, this study established a complete and self‐consistent chain of evidence spanning from material design to cellular regulation, and from in vitro organoids to in vivo animal models. Specifically, JPS mimicked the bilayered structure of the periosteum, and the synergy between its piezoelectric effect and drug release may confer dual regulatory capabilities in both vascular and osteogenic processes. Organoid experiments demonstrated that JPS may precisely modulate the vascular microenvironment of bone formation, with the formation of H‐type vascular subtypes potentially playing a central role in osteogenesis. These H‐type vessels may not only help improve nutrient transport challenges, but also influence the differentiation and maturation of surrounding osteoprogenitor cells through paracrine signaling from their endothelial cells [41]. Animal experiments further supported the coupled processes of angiogenesis and osteogenesis. In the genetically diabetic db mouse calvarial defect model, the JPS group showed markedly superior repair outcomes compared to the other groups.

### 3.10 Limitations and Perspectives

One limitation of the present study lies in the use of simplified in vitro models. Specifically, the diabetic condition was modeled using a high‐glucose environment (25 mm) relative to a normal‐glucose control (5.5 mm), and the HG‐only group was defined as the diabetic baseline control throughout the manuscript. Although this model is widely adopted to mimic diabetic stress, it does not fully capture the complexity of the in vivo diabetic microenvironment, including systemic metabolic disturbances and inflammatory cues. In addition, HUVECs were employed as a standard endothelial model to evaluate angiogenic behavior. While this model is suitable for assessing general endothelial responses, it does not represent bone‐specific endothelial cells and therefore cannot fully recapitulate the biological identity of type H vessels. To address this limitation, conclusions regarding type H vessel formation were not based solely on HUVEC assays, but were primarily supported by scRNA‐seq analysis of the diabetic bone microenvironment, CD31/EMCN‐positive vascular features observed in vascular organoids, and in vivo immunofluorescence evidence. Nevertheless, further validation using primary bone‐derived endothelial cells would strengthen the tissue specificity of these findings.

At the in vivo level, another limitation is that the local micromechanical environment at the defect site, as well as the corresponding piezoelectric output, was not directly measured or simulated. As a result, the contribution of piezoelectric stimulation was inferred from in vitro piezoelectric characterization in combination with the observed regenerative outcomes, rather than being quantitatively validated in situ. Future studies integrating mechanical modeling and in vivo piezoelectric measurement will be necessary to further clarify this aspect.

## 4 Conclusion

In the present study, a periosteum‐inspired Janus scaffold with piezoelectric properties was developed to enhance the therapeutic efficacy of diabetic bone repair. The scaffold featured a bilayered configuration: the upper layer was a zinc‐doped, apatite–gelatin–PLLA piezoelectric membrane fabricated using electrospinning, whereas the lower layer consisted of a resveratrol‐loaded, microsphere, composite hydrogel. The two layers were chemically crosslinked using genipin to achieve stable and adaptive interfacial integration. JPS not only exhibited good piezoelectric performance and biocompatibility. Mechanistically, JPS was associated with the upregulation of angiogenesis‐related signaling and with improved endothelial metabolic phenotype under high‐glucose conditions. In the genetically diabetic db mouse calvarial defect model, the bone volume fraction (BV/TV) in the JPS group was 2.48 times that in the control group, reaching 21.1%. Overall, this study presents an effective and robust material fabrication strategy to address the clinical challenges of pathological bone defects in a diabetic microenvironment relevant to impaired bone–vascular regeneration.

## Author Contributions

K. W. was responsible for the planning, manuscript writing, execution, data collection, and analysis of the biological part of this experiment. K. J. was responsible for the design, implementation, data collection, manuscript writing, and processing of the material part of this experiment. K. J. and K. W. jointly raised funds. All the authors edited and approved the final paper.

## Conflicts of Interest

The authors declare no conflicts of interest.

## Supporting information




**Supporting File**: advs75948‐sup‐0001‐SuppMat.docx.

## Data Availability

The data that support the findings of this study are available from the corresponding author upon reasonable request.
