# Supplementary material for: A Periosteum‐Inspired Janus Piezoelectric Scaffold Using Bioenergetic‐Driven H‐Type Vascularization for Diabetic Bone Regeneration
Source: Adv Sci (Weinh). 2026 Jun 4:e75948. Online ahead of print. doi: 10.1002/advs.75948 (PMC13336832; doi:10.1002/advs.75948)
Supplement: Supplementary file 1 — Supporting File: advs75948‐sup‐0001‐SuppMat.docx. [file ADVS-9999-e75948-s001.docx]

**Supporting Information**

**A Periosteum-Inspired Janus Piezoelectric Scaffold using Bioenergetic-Driven H-type Vascularization for Diabetic Bone Regeneration**

Kai Wang (王凯) ^a^, Kai Jiang (江凯) ^b,^ *

^a^ Department of Spine Surgery, Honghui Hospital, Xi'an Jiaotong University, Xi'an, Youyidong Road, Shanxi, 710054, China

^b^ Department of Critical Care Medicine, Sichuan Provincial People's Hospital, University of Electronic Science and Technology of China, Chengdu 611731, China

^^[[1]](#footnote-1)^^

**Table S1.** Ion concentrations in prepared 4×SBF mediums.

| Ions (mM) | Cl^−^ | Na^+^ | Ca^2+^ | HPO_4_^2-^ | K^+^ | HCO_3_^−^ | Mg^2+^ | SO_4_^2−^ |
| --- | --- | --- | --- | --- | --- | --- | --- | --- |
| 4×SBF | 591.2 | 568 | 10 | 4 | 20 | 16.8 | 6 | 2 |

**Table S2**. Primer sequences for qRT-PCR.

| **ID** | **primer name** | **primer sequence (5'to3')** |
| --- | --- | --- |
| 1 | VEGF-F | ATCGAGTACATCTTCAAGCCAT |
| 1 | VEGF-R | GTGAGGTTTGATCCGCATAATC |
| 2 | HIF-1α-F | GACCTGTGTGAGATCGACCA |
| 2 | HIF-1α-R | GTTGGTTTGGACGCCACTTC |
| 3 | PDGF-F | CCGTAACACATTTAGAAGCCAG |
| 3 | PDGF-R | CATCAAGCTACAACTTCAAGCA |
| 4 | CD31-F | CGTTGTCATTGGAGTGGTCAT |
| 4 | CD31-R | GAGTCGTAATGGCTGTTGGTT |
| 5 | VEGF-F | CTGCTGTAACGATGAAGCCCTG |
| 5 | VEGF-R | GCTGTAGGAAGCTCATCTCTCC |
| 6 | OPN-F | CCAGCCAAGGACCAACTACA |
| 6 | OPN-R | AGTGTTTGCTGTAATGCGCC |
| 7 | OCN-F | GGCGCTACCTGTATCAATGG |
| 7 | OCN-R | GTGGTCAGCCAACTCGTCA |
| 8 | Runx-2-F | CCGAGACCAACCGAGTCATTTA |
| 8 | Runx-2-R | AAGAGGCTGTTTGACGCCAT |
| 8 | COL-1-F | CCCAGCGGTGGTTATGACTT |
| 8 | COL-1-R | TCGATCCAGTACTCTCCGCT |
| 9 | GAPDH-F | CCGAGACCAACCGAGTCATTTA |
| 9 | GAPDH-R | GATGGTGATGGGTTTCCCGT |


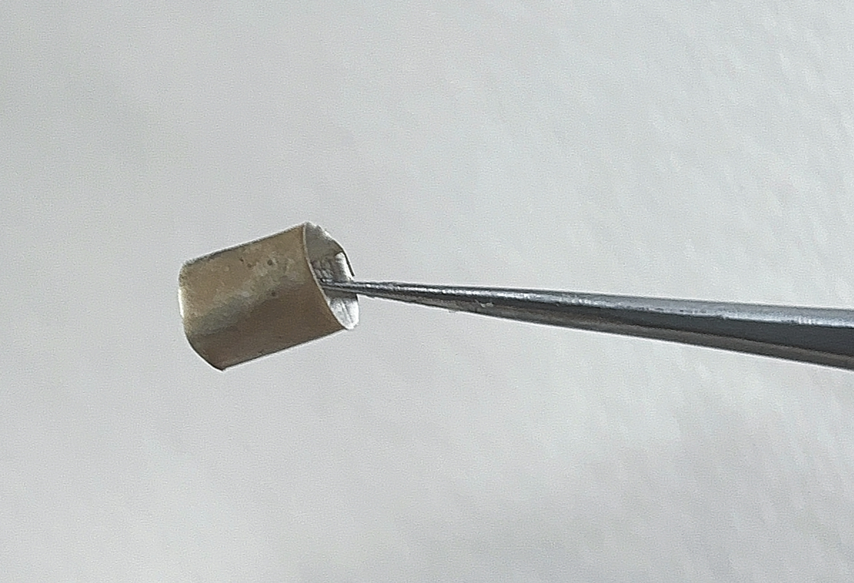


**Figure S1.** Digital photos of JPS scaffolds.

**
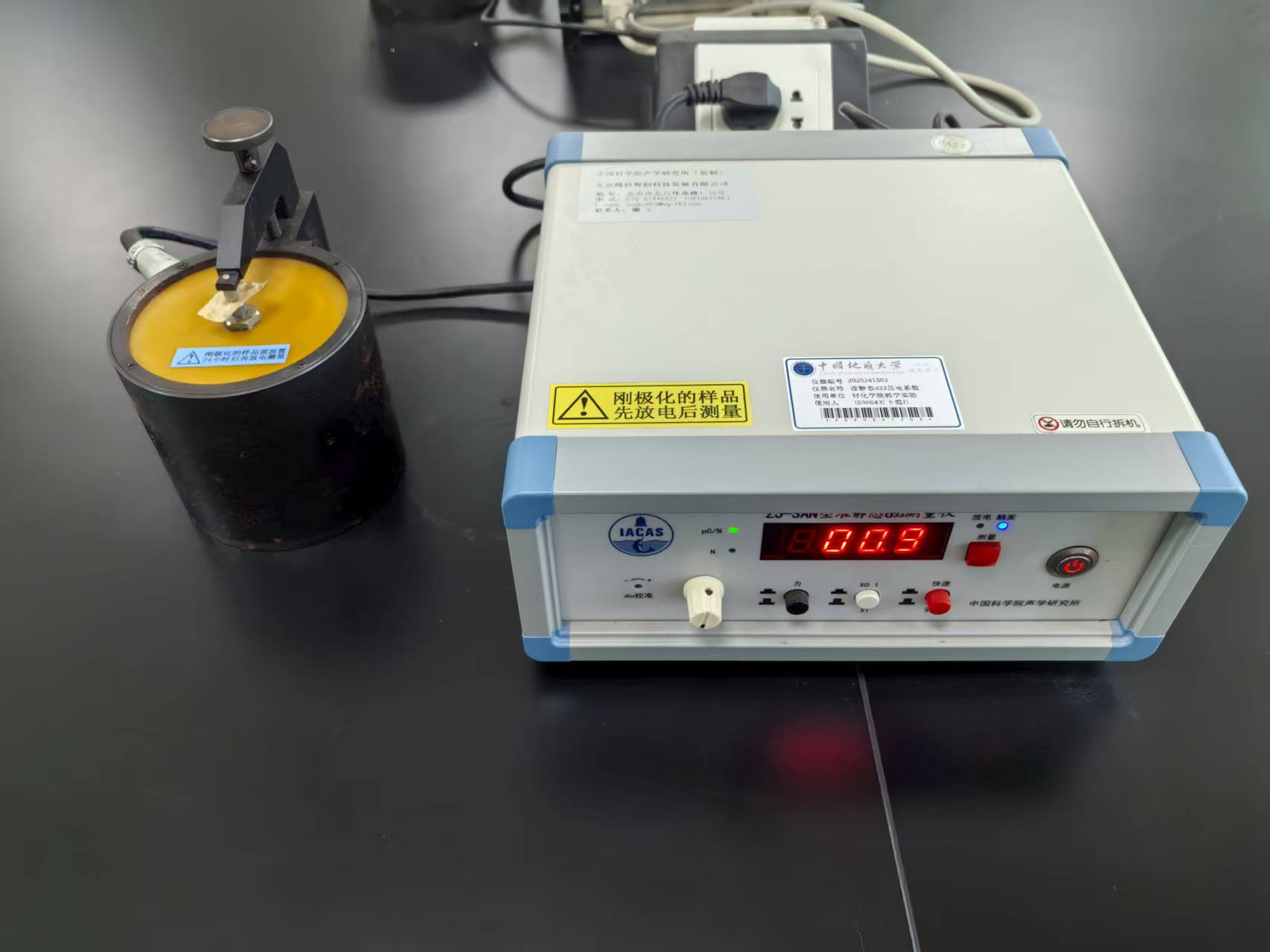
**

**Figure S2.** Digital photos of the piezoelectric constants (d33) testing of JPS.


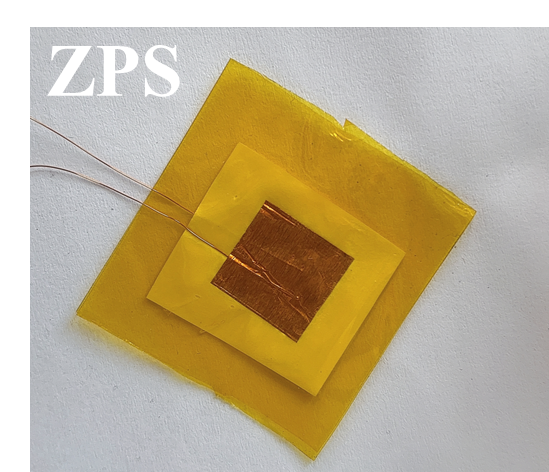


**Figure S3.** Digital photos of the encapsulated ZPS piezoelectric device.

1. First author: Kai Wang

   * Corresponding author: Kai Jiang

   E-mail addresses: mr.jiangkai@foxmail.com (K. J.) [↑](#footnote-ref-1)
